# Obesity accelerates age-related memory deficits and alters white matter tract integrity in Ldlr-/-.Leiden mice

**DOI:** 10.1016/j.bbih.2025.100991

**Published:** 2025-04-15

**Authors:** Florine Seidel, Martine C. Morrison, Ilse Arnoldussen, Vivienne Verweij, Joline Attema, Christa de Ruiter, Wim van Duyvenvoorde, Jessica Snabel, Bram Geenen, Ayla Franco, Maximilian Wiesmann, Robert Kleemann, Amanda J. Kiliaan

**Affiliations:** a Department Medical Imaging, Anatomy, Radboud Alzheimer Center, Donders Institute for Brain, Cognition, and Behavior, Radboud University Medical Center, Geert Grooteplein 21N, 6525 EZ, Nijmegen, the Netherlands; b Department of Metabolic Health Research, Netherlands Organisation for Applied Scientific Research (TNO), Sylviusweg 71, 2333 BE, Leiden, the Netherlands

**Keywords:** Aging, Obesity, Cognitive impairment, Neurodegeneration, Neuroimaging

## Abstract

**Background:**

Obesity in mid-adulthood has been suggested to promote brain aging and is associated with progressive cognitive impairment later in life. However, the structural and functional alterations that underlie obesity-related cognitive dysfunction are still poorly understood, partly owing to the lack of translational models replicating age- and obesity-related brain pathology.

**Methods:**

The effect of age and high-fat diet (HFD)-induced obesity was investigated in adult Ldlr-/-.Leiden mice, an established translational model for obesity and its comorbidities. During mid-adulthood, from three to eight months of age, brain structure and function (hippocampal volume, cortical thickness, white matter integrity, cerebral blood flow (CBF), resting-state functional connectivity) were monitored with brain magnetic resonance imaging, and cognitive function was evaluated using cognitive tests. Brain pathology was further examined with histopathological and gene expression analyses.

**Results:**

Ldlr-/-.Leiden mice showed age-related decreases in cortical thickness, CBF, brain connectivity, and neurogenesis along with the development of neuroinflammation and (short-term) memory impairments. On HFD feeding, Ldlr-/-.Leiden mice exhibited similar features, but memory deficits started at a younger age than in chow-fed mice. HFD-fed mice additionally showed a rise in CBF with concomitant decline in fractional anisotropy in white matter tracts. Analyses of hippocampal gene expression further revealed an age-related suppression of processes related to metabolic and neuronal function while HFD feeding strongly activated neuroinflammatory pathways.

**Conclusions:**

Ldlr-/-.Leiden mice show similar critical age-related changes in brain structure and function as observed in humans. In this mouse model, HFD feeding particularly trigger disturbances in brain blood perfusion and white matter tract integrity, which may underlie an accelerated cognitive decline in obesity.

## 1 Introduction

Obesity is a global health concern that has reached alarming proportions over the past few decades (Blüher, 2019). It is widely recognized that obesity is linked to numerous comorbidities including type II diabetes, hypertension, cardiovascular diseases and certain types of cancer (Guh et al., 2009). More recently, it has been demonstrated that obesity in mid-adulthood not only increases metabolic imbalances but also readily compromises brain integrity in a manner similar to aging. As observed in elderly persons, middle-aged adults with high body mass index (BMI) exhibit detrimental alterations of brain structure such as cortical thinning (Shaw et al., 2018; Frangou et al., 2022) and reduced total brain and grey matter volumes (Scahill et al., 2003; Dekkers et al., 2019). Both obesity and normal aging are additionally associated with decreased white matter integrity. White matter integrity can be assessed with diffusion tensor imaging (DTI) in which fractional anisotropy (FA) indexes provide quantitative measurement of fiber orientation in white matter tracts and may reflect fiber density, axonal diameter and the degree of myelination (Mori and Zhang, 2006). In both aging adults and people living with obesity, lower FA in white matter tracts has been described, indicating significant disruption of white matter microstructure (Westlye et al., 2010; Stanek et al., 2011).

Along with structural alterations, normal aging and obesity are associated with a global alteration in brain functional connectivity at rest, and, notably, they share a common decrease in connectivity within the default-mode network (Syan et al., 2021; Ferreira and Busatto, 2013). In parallel, reductions in cerebral blood flow (CBF), which are associated with increasing risk of dementia (Wolters et al., 2017), have been observed (Amen et al., 2020; Martin et al., 1991). All these studies highlight the common vulnerability of the hippocampus, a key structure involved in learning and memory, to deleterious processes related to neurodegeneration (Bartsch and Wulff, 2015). In particular, both age and BMI have been associated with hippocampal shrinking (Scahill et al., 2003; Cherbuin et al., 2015) together with decreased synaptic plasticity, as well as vascular and inflammatory alterations (Bettio et al., 2017; Lee and Yau, 2020). In other studies, aging and obesity have been demonstrated to be separate risk factors, each contributing to the progressive decline in cognitive performance (Sellbom and Gunstad, 2012; Murman, 2015). Altogether, these findings suggest that obesity may accelerate brain aging. This is further supported by volumetric studies estimating a 10-year increase in brain age in middle-aged individuals with obesity (Ronan et al., 2016). Despite the growing understanding of the effects of aging and obesity on cognitive function, there is insufficient understanding on how aging and obesity interact at the structural, functional, and molecular levels in order to develop effective strategies for prevention and treatment. This may, in part, be due to the lack of well-characterized translational models reflecting simultaneous attributes of age and obesity-related brain alterations.

This study therefore aims to delineate age- and obesity-related alterations of brain structure and function in mid-adulthood using Ldlr-/-.Leiden mice. Ldlr-/-.Leiden mice, an established pre-clinical model for obesity and associated vascular and metabolic complications, have been shown to develop comorbidities akin to humans by replicating human disease processes on molecular level (van den Hoek et al., 2020; Verschuren et al., 2024; Morrison et al., 2018). When these mice are fed an energy-dense high-fat diet (HFD) with a macronutrient composition similar to a human diet, they exhibit obesity, insulin resistance and dyslipidemia (van den Hoek et al., 2020; Morrison et al., 2018). They also gradually develop white adipose tissue inflammation, increased gut permeability and vascular disease (atherosclerosis) (Seidel et al., 2022; Gart et al., 2022, 2023). Using histological and gene expression analyses, we have previously shown that Ldlr-/-.Leiden mice on chow diet are prone to develop neurodegeneration and age-related astrogliosis, which was not observed in wildtype (C57BL6/J) mice treated the same way (Seidel et al., 2023). The aim of the present study was to characterize the structural and functional brain alterations that occur during the early phase of aging (referred to as incipient aging) and the effects of obesity on these processes. To do so, we performed *in vivo* brain magnetic resonance imaging (MRI), cognitive tests, immunohistochemical analyses and hippocampal gene expression analyses in young-adult mice on a regular chow diet (3-month-old young lean reference) and in mature-adult mice on chow diet or on HFD. This enabled us to investigate whether age-related processes are aggravated by obesity, and to eventually identify processes that are exclusively driven by obesity. Our study demonstrates that Ldlr-/-.Leiden mice replicate key processes of age- and obesity-related brain impairment observed in humans, which substantiates the use of this model as a valuable translational tool to investigate cognitive decline, both in the context of normal aging and obesity. Our findings provide new insights into the structural and functional alterations that may underlie the initial phase of aging-related cognitive dysfunction and the additional detrimental effects of obesity on these processes.

## 2 Materials and methods

### 2.1 Study design and animals

**Fig. 1:**
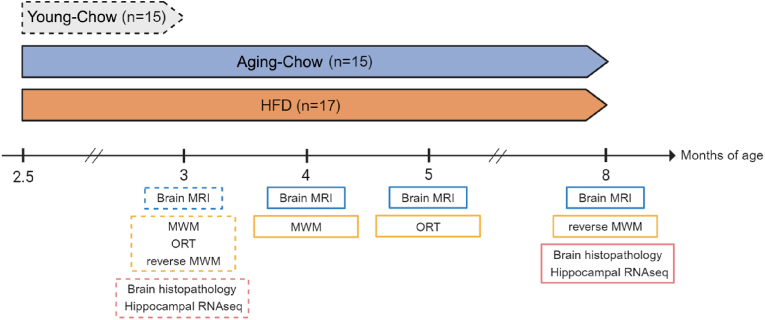
**Experimental design.** At the start of the study the animals were 10–12 weeks old (∼2.5 months of age corresponding to young adults). One group of chow-fed mice (Young-Chow, n = 15) was euthanized at 3 months of age as a young lean (healthy) reference. From 2.5 to 8 months of age, one aging group (Aging-Chow, n = 15) remained on a standardized chow diet and another aging group (HFD, n = 17) was fed an obesity-inducing energy-dense high-fat diet (HFD). At 3 months of age for the Young-Chow group (dotted lines) and at 4, 5 and 8 months of age for the Aging-Chow and HFD groups (solid lines), brain MRI was performed to assess brain structure (cortical thickness, hippocampus size, white matter integrity) and brain function (CBF and vasoreactivity, rs-FC) and behavioral tests ((reverse)Morris Water Maze (MWM) and Object Recognition Test (ORT)) were conducted to assess short-term memory and spatial learning. At sacrifice, the brains were collected for histopathological analyses and hippocampal RNA sequencing (RNAseq).

47 male Ldlr-/-.Leiden mice were obtained from an internal breeding stock of TNO Metabolic Health Research (Leiden, the Netherlands) and housed in the Pre-clinical Imaging Center (PRIME) of Radboud university medical center (Nijmegen, the Netherlands). The animals in this study are part of a larger study which is comparable to a previous longitudinal study in C57BL/6 J mice (Mulder et al., 2016), and approved by an independent Animal Welfare Body (approval number TNO-458) and the Veterinary Authority of Radboud university medical center (approval number 2017-0063-004). All procedures performed on these animals were in accordance with European Union regulations and guidelines on animal research. All animals were group-housed (2–4 mice per cage) in Digitally Ventilated Cages (Techniplast SPA, Buguggiate, Italy) and cages from different groups were randomized over shelves in a clean-conventional animal room (relative humidity 50–60 %, temperature 21 °C, 12-h light/dark cycle). The animals received unlimited food and water throughout the study. Until the start of the study all mice were fed a standardized chow diet (Sniff R/M-H diet, Sniff Spezialdiäten GmbH, Soest, Germany). An overview of the experimental design is provided in Fig. 1. At the start of the experiment the animals were 10–12 weeks old (∼2.5 months of age corresponding to young adults). One group of chow-fed mice (Young-Chow, n = 15) was euthanized at 3 months of age as a younger lean (healthy) reference. Then, from 2.5 to 8 months of age, one aging group (Aging-Chow, n = 15) remained on the standardized chow diet and another aging group (HFD, n = 17) was fed an obesity-inducing energy-dense high-fat diet (HFD, D12451, Research Diets, New Brunswick, NJ, USA). The group that ages on a chow diet will hereafter be referred to as the ‘normal aging’ group. Food intake and individual body weights were recorded weekly, and 5 h-fasted blood samples were collected via the tail artery at 2.5, 4, 5 and 8 months of age. Blood glucose, plasma cholesterol and triglycerides were measured as previously described (Seidel et al., 2022). At 3 months of age for the Young-Chow group and at 4, 5 and 8 months of age for the Aging-Chow and HFD groups, brain MRI and behavioral tests were performed. At 3 months of age for the Young-Chow group or at 8 months of age for the Aging-Chow and HFD groups, mice were euthanized by cervical dislocation after transcardial perfusion with phosphate-buffered saline at room temperature.

### 2.2 Brain MRI

Brain MRI was performed using an 11.7 T BioSpec Avance III small animal MR system with Paravision 6.0.1 software (both Bruker Biospin, Ettlingen, Germany). All MRI procedures are described in detail in the Supplementary material. Briefly, isoflurane (3.5 % for induction, 1.8 % for maintenance) in a 1:2 oxygen-air mixture was used to anesthetize the animals. Anatomical images (T2-weighted) were acquired to measure the hippocampal volume and the thickness of cortical regions (i.e. auditory, motor, somatosensory and visual cortices). DTI was used to measure FA and mean diffusivity in white matter (i.e. corpus callosum, optic tract, fornix) and grey matter (i.e. hippocampus, basal ganglia (caudate nucleus, putamen and globus pallidus) and auditory, motor, somatosensory and visual cortices). An arterial spin labeling sequence with flow-sensitive alternating inversion recovery (FAIR) method was used to measure CBF (Wiesmann et al., 2017). CBF was first measured under 1:2 oxygen – air mixture (normal gas conditions) and under pure oxygen (vasoconstrictive gas conditions) in the cortex, hippocampus, and thalamus. Vasoreactivity, defined as the capacity of the blood vessels to constrict upon stress conditions, was obtained by subtracting CBF under normal gas conditions from CBF under vasoconstrictive gas conditions divided by CBF under normal gas conditions. Finally, resting-state functional MRI was employed to measure intra and interhemispheric resting-state functional connectivity (rs-FC) between brain regions critical for cognitive function (i.e. dorsal and ventral hippocampus, auditory, somatosensory, motor, and visual cortex). Total correlations were used to determine total connectivity between two brain regions while partial correlations were used to determine direct relationships between two brain regions independently of other brain regions.

### 2.3 Cognitive tests

#### 2.3.1 (reverse) Morris Water Maze ((r)MWM)

Cognitive tests to assess hippocampus-dependent cognitive function (e.g., spatial learning, short-term memory) were performed at multiple time points. To avoid habituation and minimize possible learning effects, a different test was performed at each time point. A MWM test was performed in the Young-Chow group at 3 months of age and in the Aging-Chow and HFD groups at 4 months of age, to assess spatial learning and short-term memory. Briefly, during a first 4-day acquisition phase (learning phase) the mice were trained to find a submerged platform below the water surface (made opaque with milk powder) located in the North-East quadrant using distant visual cues. The mice performed four trials per day, starting respectively from the south, east, north and west wall of the pool. The average velocity, escape latency (time to find the platform) and distance swam before finding the platform were monitored with Ethovision XT (v15, Noldus, Wageningen, the Netherlands) for each trial and each parameter was averaged per day. At the end of the acquisition phase, the platform was removed and the mice were allowed to swim during 2 min during which the average velocity and time spent in the North-East quadrant or in the platform zone was recorded (probe phase).

A reverse MWM (rMWM) test was performed at 3 months of age in the Young-Chow group (4 days after the MWM test) and at 8 months of age in the Aging-Chow and HFD groups. Using a similar procedure to that of the MWM, a 2-day acquisition phase was conducted during which the mice were trained to find a submerged platform located this time in the South-West quadrant of the pool. A final probe test was performed during which the platform was removed and the average velocity, time spent in the South-West quadrant or in the platform zone were recorded during 2-min swimming.

During the acquisition phase of the (r)MWM, cognitive scores were determined for each trial based on the type of strategy using the Pathfinder program (Cooke et al., 2019). Cognitive scores were attributed as followed (Curdt et al., 2022): 0: thigmotaxis, 1: random search, 2: scanning, 3: chaining, 4: indirect search or semi-focal search or focal search, 5: directed search, 6: direct path. Scores 4–6 were considered hippocampus-dependent search strategies. Cognitive scores were averaged per day and the percentage of trials using hippocampus-dependent strategy were determined per day.

#### 2.3.2 Object Recognition Test (ORT)

An ORT was performed at 3 months of age in the Young-Chow group and at 5 months of age in the Aging-Chow and HFD groups, to characterize exploration behavior and short-term memory. The ORT included two distinct phases: a familiarization phase during which the mice were allowed to explore two similar objects during 2 min, and a test phase during which the mice were allowed to explore one former object of the familiarization phase and one novel object. For both phases, the total exploration time and the separate exploration time for both objects were automatically detected in Ethovision. ‘Short-distance exploration’ included exploration by touch and smell of the objects (i.e., nose-point located within a 2-cm diameter around the object) while ‘visual exploration’ also included visual inspection of the objects (i.e., head directed towards the objects). The ORT was repeated during three days and the time between the familiarization and test phases was respectively 30 min, 1 h and 2 h. The type of objects and the position of the novel objects (i.e. left or right) were randomized and balanced across the trials. For the trials of the test phase, a recognition index was calculated as the percentage of time spent exploring the novel object relative to the total exploration time. A discrimination index was also calculated as the exploration time of the familiar object subtracted to the exploration of the novel object and divided by the total exploration time. A positive value indicates longer exploration of the novel object while a negative value indicates longer exploration of the familiar object. For the calculations of these indexes, mice that did not explore the objects during the familiarization phase were excluded for the corresponding test phase, and the animals that did not explore the objects during the test phase were attributed a value of 0 for both the discrimination and recognition indexes.

### 2.4 Immunohistochemical analyses

#### 2.4.1 Brain pathology

Tissue preparation and immunohistochemial procedures were conducted as reported previously (Lohkamp et al., 2023). 30 μm-thick free-floating coronal cross-sections of brain left hemispheres were stained for doublecortin (DCX) as a marker for neurogenesis, for ionized calcium-binding adapter molecule 1 (IBA-1) as a marker for microglia activation, for glial fibrillary acidic protein (GFAP) as a marker for astrogliosis, and for glucose transporter 1 (GLUT-1) as a marker for blood capillaries. Antibodies used and corresponding dilutions are listed in Supplementary Table S1. Sections were scanned at 20x magnification (Aperio AT2, Leica, Amsterdam, the Netherlands). DCX-positive neurons were manually counted in the hippocampus by two independent, blinded examiners using up to three consecutive non-overlapping cross-sections. For IBA-1 and GLUT-1 staining, the total positive area and number of positive particles were analyzed in white matter (corpus callosum and fimbria) and grey matter (cortex, hippocampus, and thalamus). For GFAP staining, the total positive area and the staining intensity were determined in grey matter areas (i.e. cortex, hippocampus, and thalamus). For IBA-1, GLUT-1 and GFAP staining, up to three consecutive non-overlapping cross-sections (bregma ∼ -1.94) were used and results were averaged for each brain region. An automated quantification including intensity-based threshold to distinguish positive particles from background staining was performed with ImageJ (v1.51, National Institutes of Health, United States).

#### 2.4.2 Atherosclerosis

Atherosclerosis was analyzed as the total number of lesions, total lesion size and average lesion size in 5 μm-thick cross-sections of the aortic roots stained with hematoxylin-phloxine-saffron as previously described (Seidel et al., 2022).

### 2.5 Hippocampal gene expression and pathway analysis

Brain tissue was collected in 1 ml cold Trizol (Invitrogen, Paisley, UK) and homogenized using a dispersing machine (ultra-Turrax; IKA Werke GmbH & Co. KG, Staufen, Germany) as previously described (Janssen et al., 2015). After chloroform extraction and isopropyl alcohol precipitation, total RNA was dissolved in 25 μl RNase-free diethylpyrocarbonate (DEPC)-treated water. The RNA concentration of the tissue was measured with a Nanodrop 1000 spectrophotometer (Thermo Fisher Scientific Inc, Wilmington, DE, USA). mRNA was isolated from total RNA with oligo-dT magnetic beads. Next, cDNA was synthesized and subsequently ligated with sequencing adapters and amplified by PCR and was processed into tagged random sequence libraries (NEBNext Ultra II Directional RNA Library Prep Kit for Illumina, NEB #E7760S/L , Biolabs) and sample quality was checked for proper size distribution (300-500 bp peak, Fragment Analyzer). The mixed (multiplex) sample libraries were sequenced on an Illumina NovaSeq6000 v1.5 sequencer (Illumina, San Diego) with a paired-read 150-cycle sequencing protocol at GenomeScan BV (Leiden, the Netherlands), resulting in ∼20-70 million read counts per sample. Clustering and DNA sequencing using the NovaSeq6000 was performed according to manufacturer's protocols. A concentration of 1.1 nM of DNA was used yielding paired end reads (2x 150bp). NovaSeq control software NCS v1.7 was used. Image analysis, base calling, and quality check was performed with the Illumina data analysis pipeline RTA3.4.4 and Bcl2fastq v2.20. Quality filtering and adapter-trimming of the Illumina NovaSeq6000 fastq files was performed by TRIMMOMATIC software. Trimmed Fastq files were merged (in case of Paired-end reads) and aligned to the ensembl reference genome Mus_musculus.GRCm38.gencode.vM19 using the STAR 2.5 algorithm with default settings (https://github.com/alexdobin/STAR). Based on the mapped read locations and the gene annotation, Htseq-count 0.6.1p1 was used to count the read mapping frequency/gene (transcript region) resulting in count.files. The count files (.tsv) contain the # reads mapped per gene (not normalised per sample). These counts serve as input for the statistical analysis using DEseq2 package (https://bioconductor.org/packages/release/bioc/html/DESeq2.html). Differentially expressed genes were used for pathway and upstream regulator analyses using Ingenuity Pathway Analysis (IPA; www.ingenuity.com, accessed on 2 October 2022) as previously detailed (Gart et al., 2021, Krämer et al., 2014). For these analyses, a cut-off of p-value (P)<0.01 was used. When possible, a z-score was calculated for which a positive value ≥ 2 indicates relevant activation and a negative value ≤ -2 a relevant inhibition of the canonical pathway or upstream regulator. The RNA-seq dataset obtained for this study is publicly available in the Gene Expression Omnibus (GEO) repository (https://www.ncbi.nlm.nih.gov/gds, accession number: GSE294054).

### 2.6 Statistical analyses

Data are expressed as mean ± standard deviation (SD). Statistical power analyses and sample size calculations were performed based on the MWM and MRI arterial spin labeling (f = 0.10) to minimize the number of animals used in the experiment. Datasets were examined for outliers, and significant outliers were excluded if technical or biological anomalies were detected. Statistical analyses were performed with SPSS software version 28 (IBM, Armonk, NY, USA). Normal distribution of the variables and homoscedasticity assumptions were examined with a Shapiro-Wilk test and Levene's test respectively. When variables were not normally distributed, a transformation according to the Tukey ladder of powers was performed or, when transformations were not possible, non-parametric tests were used. In a first analysis, intragroup changes over time and intergroup comparisons were examined between Aging-Chow and HFD groups using repeated measures general linear model (GLM) with Bonferroni correction for multiple testing or using univariate/multivariate GLM when only one time point was available (e.g. histological analyses). In a separate analysis, Aging-Chow and HFD groups at different ages were compared with the Young-Chow group using univariate/multivariate GLM followed by a Dunnett post-hoc with the Young-Chow group as reference. Finally, Spearman's correlations were used to explore associations between parameters related to brain structure and function (e.g., cortical thickness, hippocampal volume, fractional anisotropy, brain perfusion, and rs-FC) and cognitive parameters (e.g., (r)MWM probes, ORT discrimination, and recognition indices). P-values lower than 0.05 were considered statistically significant. An overview of the p-values for each analysis is available in Supplementary Tables S2–S12.

## 3 Results

### 3.1 Upon HFD feeding, Ldlr-/-.Leiden mice develop obesity and associated comorbidities

**Fig. 2:**
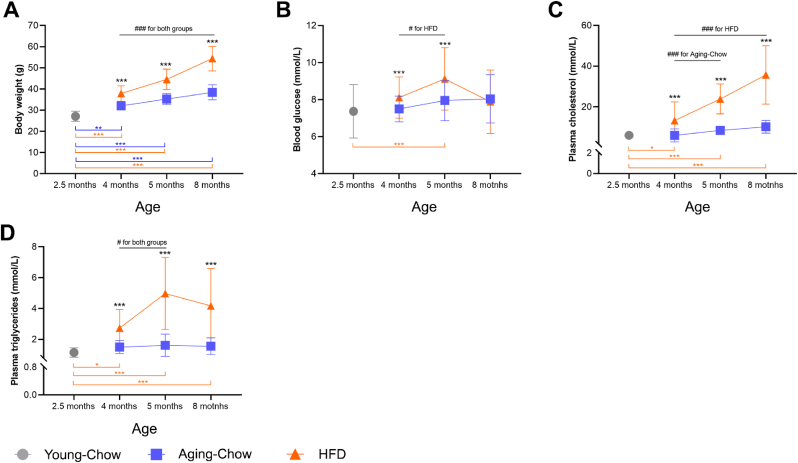
**Body weight and metabolic health.** (A) body weight, (B) blood glucose, (C) plasma cholesterol and (D) plasma triglyceride contents in Young-Chow (3 months old) and over aging in Aging-Chow and HFD groups. Data are shown as mean ± SD. #p < 0.05, ###p < 0.001 for intragroup effects over time; ∗p < 0.05, ∗∗p < 0.01, ∗∗∗p < 0.001 for intergroup effects.

When compared with Aging-Chow mice, HFD-fed Ldlr-/-.Leiden mice exhibited substantial increase in body weight, hypercholesterolemia and hypertriglyceridemia, abnormal blood glucose levels (Fig. 2) and severe atherosclerosis (Fig. S1). The Aging-Chow group also gained weight over time, displaying significantly higher body weight compared with the 3-month-old Young-Chow group. By 8 months of age, they also exhibited mild forms of atherosclerosis.

### 3.2 Ldlr-/-.Leiden mice show age-related cortical thinning

**Fig. 3:**
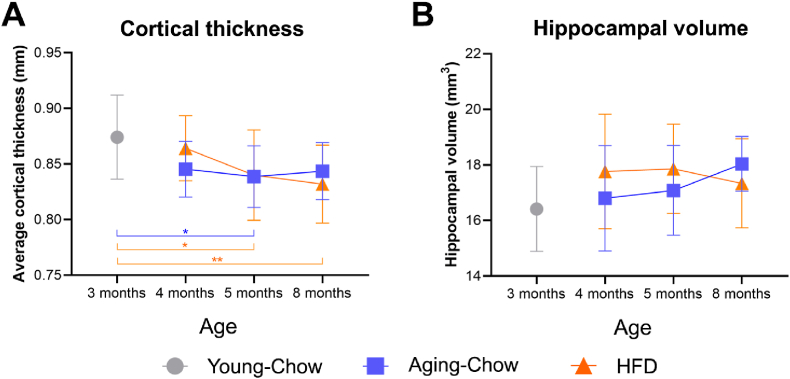
**Cortical thickness and hippocampal volume.** (A) Average cortical thickness and hippocampal volume were measured on T2-weighted anatomical images. Data are shown as mean ± SD. ∗p < 0.05, ∗∗p < 0.00.01 for intergroup effects.

Hippocampal volume and cortical thickness were measured using T2-weighted brain images. The average cortical thickness did not change over time in the Aging-Chow and HFD groups (p = 0.085, Fig. 3A) and all the groups had comparable cortical thickness until 4 months of age. At 5 months of age, both the Aging-Chow group and HFD group exhibited on average a thinner cortex than the 3-month-old Young-Chow mice. At 8 months of age, only the HFD group had a smaller cortical thickness than the 3-month-old Young-Chow mice. No differences in cortical thickness were observed between the Aging-Chow group and the HFD group at any measured time point. The thickness of individual cortical regions that were used to calculate the average cortical thickness are available in Fig. S2. No differences in hippocampal volume were observed between the groups (Fig. 3B).

### 3.3 HFD-fed Ldlr-/-.Leiden further exhibit impaired white matter integrity

**Fig. 4:**
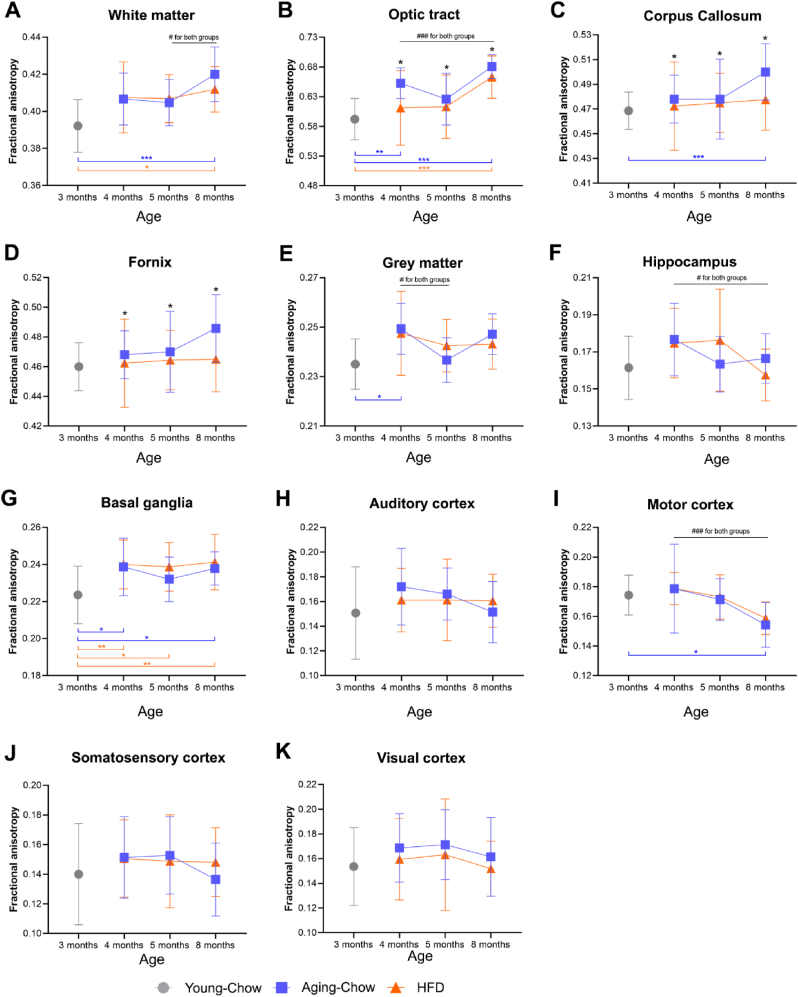
**Fractional anisotropy in white matter and grey matter.** Using DTI, fractional anisotropy (FA) was measured in (A) white matter including (B) the optic tract, (C) the corpus callosum and (D) the fornix, and in (E) grey matter including (F) hippocampus, (G) basal ganglia (caudate nucleus, putamen and globus pallidus), (H) auditory cortex, (I) motor cortex, (J) somatosensory cortex and (K) visual cortex. Data are shown as mean ± SD. #p < 0.05, ###p < 0.001 for intragroup effects over time; ∗p < 0.05, ∗∗p < 0.01, ∗∗∗p < 0.001 for intergroup effects.

White and grey matter integrity was assessed by measuring FA and mean diffusivity using DTI. In overall white matter, an increase in FA during aging in both the Aging-Chow and HFD groups was observed between 5 and 8 months of age resulting in a significantly higher FA compared with Young-Chow controls (3 months of age, Fig. 4A). This global FA increase in white matter in the Aging-Chow and HFD groups was accounted for by a FA increase in the optic tract between 4 and 8 months of age (Fig. 4B). In parallel, compared with Aging-Chow animals at all ages, HFD mice showed significantly lower FA in the optic tract (−4 %, Fig. 4B), corpus callosum (−2 %, Fig. 4C) and fornix (−2 %, Fig. 4D), possibly indicating lower fiber tract density and myelination in the HFD group.

FA in grey matter decreased between 4 and 5 months of age in both the Aging-Chow and HFD groups (Fig. 4E), and this effect can mainly be ascribed to decreases in FA in the hippocampus and motor cortex (Fig. 4F–K). No significant differences were observed between HFD and age-matched Aging-Chow mice. Besides, no differences were observed for mean diffusivity in white and grey matter, except in the optic tract for which mean diffusivity was significantly lower in HFD mice than in age-matched Aging-Chow mice (Fig. S3).

### 3.4 CBF decreases with aging but increases on HFD feeding

**Fig. 5:**
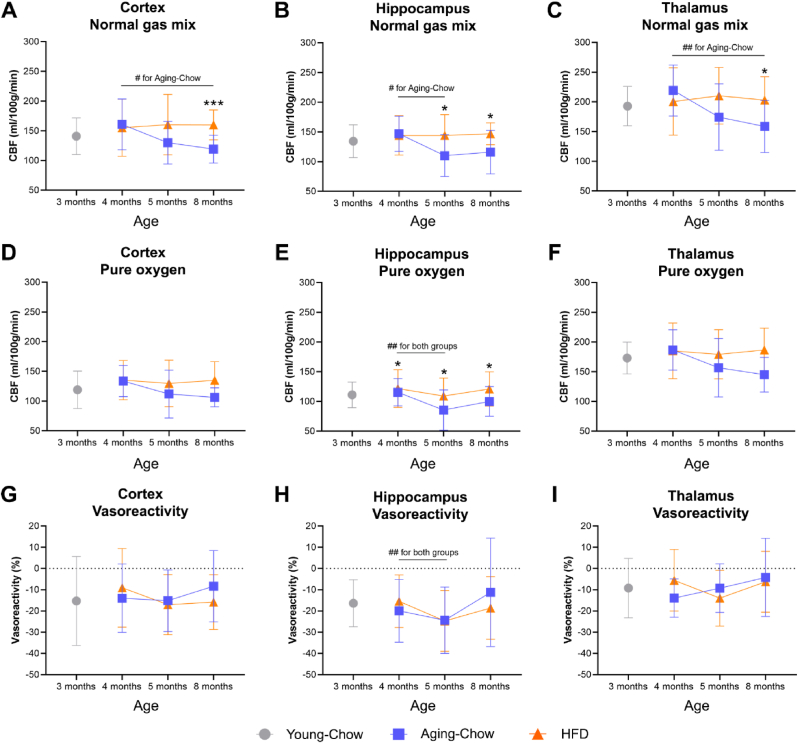
**Cerebral perfusion based on arterial spin labeling.** (A–C) Cerebral blood flow (CBF) under normal gas mix (1:2 oxygen-air) and (D–F) CBF under pure oxygen (vasoconstrictive conditions) were measured in the cortex, hippocampus and thalamus using an arterial spin labeling. (G–I) Vasoreactivity was measured as the difference between CBF under pure oxygen and CBF under normal gas mix and normalized by CBF under normal gas mix. For vasoreactivity, a lower negative value indicates higher vasoreactivity (i.e. a greater ability to constrict/dilate the vessels). Data are shown as mean ± SD. #p < 0.05, ##p < 0.01 for intragroup effects over time; ∗p < 0.05, ∗∗∗p < 0.001 for intergroup effects.

Under normal gas conditions (1:2 oxygen-air), CBF in all regions investigated (i.e., cortex, hippocampus and thalamus) decreased over time in Aging-Chow mice (Fig. 5A–C). By contrast, CBF in the HFD group remained stable over time, resulting in a significantly higher CBF in HFD mice, from 12 weeks of HFD feeding (i.e., 5 months of age) in the hippocampus, and from 27 weeks of HFD feeding (i.e., 8 months of age) in the cortex and thalamus.

Under pure oxygen-induced vasoconstrictive gas conditions, CBF remained higher in the hippocampus of HFD mice compared with age-matched Aging-Chow mice from 4 months of age onwards (Fig. 5E), but no differences were observed anymore between the groups in the cortex and thalamus (Fig. 5D–F).

Cerebral vasoreactivity was finally calculated as the difference between CBF measured in normal and vasoconstrictive conditions, and no group differences were detected for any of the brain regions analyzed (Fig. 5G–I). An increase in vasoreactivity (lower negative value) was only observed in the hippocampus in HFD and Aging-Chow mice between 4 and 5 months of age.

### 3.5 HFD feeding accelerates the age-related reduction in brain connectivity

**Fig. 6:**
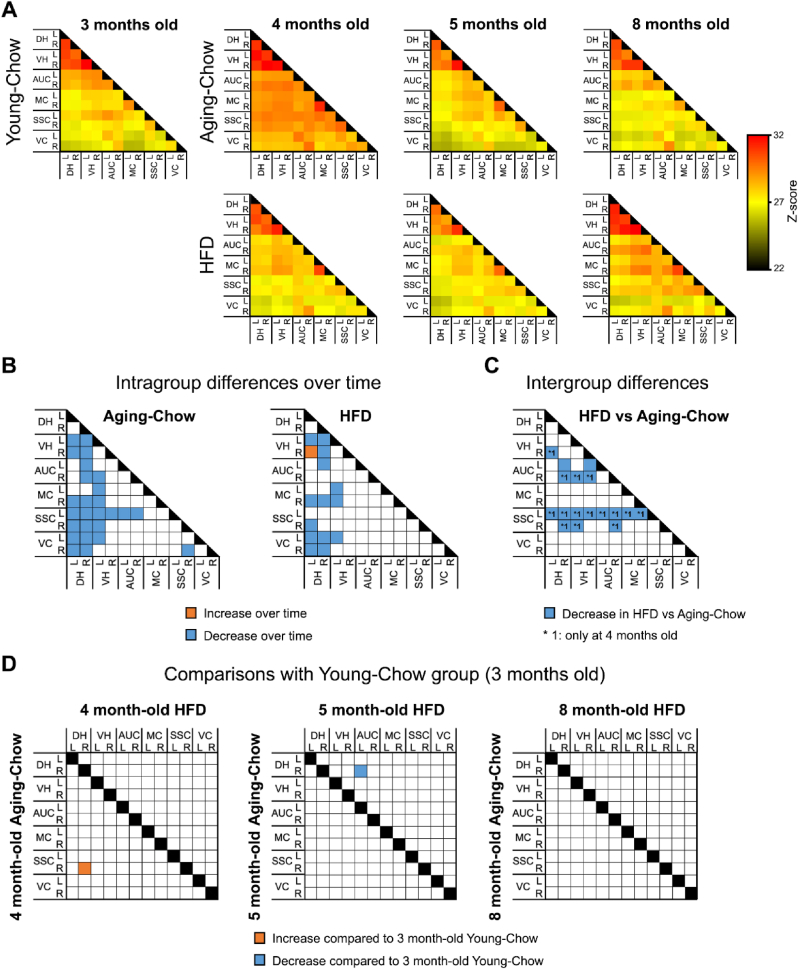
**Rs-FC based on total correlations in resting-state functional MRI.** (A) Heatmaps showing total correlations between brain regions in Young-Chow (3 months old), Aging-Chow and HFD groups. A higher z-score indicates stronger functional connectivity. (B) Overall intragroup changes in rs-FC between 4 and 8 months of age in Aging-Chow and HFD groups. (C) Group differences in rs-FC between HFD and Aging-Chow groups. (D) Comparison of rs-FC of 4 month-old, 5 month-old and 8-month old Aging-Chow and HFD groups with rs-FC of Young-Chow group (3 months old). Abbreviations: (DH) dorsal hippocampus; (VH) ventral hippocampus; (AUC) auditory cortex; (MC) motor cortex; (SSC) somatosensory cortex; (VC) visual cortex in left (L) and right (R) hemispheres.

Brain connectivity was assessed with resting-state functional MRI. Heatmaps showing the connectivity between brain regions based on total correlations are displayed in Fig. 6A. A global decrease in total connectivity (both intra and interhemispheric) was observed with aging in both Aging-Chow and HFD groups (Fig. 6B). Of note, in the HFD group, rs-FC between left (dorsal) and right (ventral) hippocampus increased over time. At 4 months of age, rs-FC of somatosensory and auditory cortices was already lower in HFD mice than in Aging-Chow mice (Fig. 6C). At later time points, rs-FC also significantly decreased in the Aging-Chow group so that chow-fed mice reached similar rs-FC as HFD-fed mice at 5 and 8 months of age. Compared with the Young-Chow group (3 months old), 4-month-old Aging-Chow mice showed higher functional connectivity between somatosensory cortex and dorsal hippocampus in the right hemisphere, while 5 month-old HFD mice had lower connectivity between left auditory cortex and right dorsal hippocampus (Fig. 6D).

rs-FC was also determined using partial correlations in order to estimate direct connectivity between two brain regions independently of the other brain regions, which showed several changes over time depending on the brain regions (Fig. S4).

### 3.6 Aging mice show neuroinflammation, impaired neurogenesis but increased capillary density

**Fig. 7:**
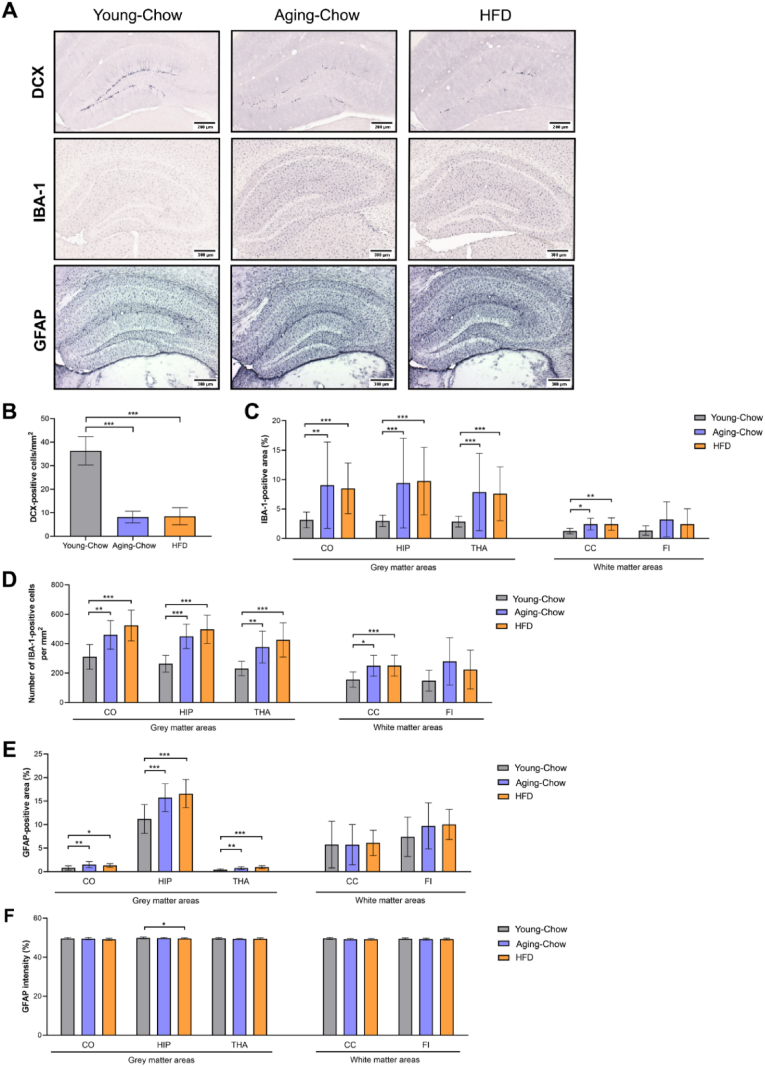
**Neurogenesis and neuroinflammation.** (A) Representative pictures of coronal brain cross-sections stained for DCX as a marker for neurogenesis, IBA-1 as a marker for microglia activation and GFAP as a marker for astrogliosis, for the Young-Chow (3 months of age), Aging-Chow (8 months of age) and HFD (8 months of age) groups. (B) Quantification of DCX-positive neurons (newly-generated neurons) in the dentate gyrus of the hippocampus. Quantification of (C) IBA-1-positive area and (D) number of IBA-1-positive cells in grey matter areas (i.e. cortex (CO), hippocampus (HIP), thalamus (THA)) and white matter areas (i.e. corpus callosum (CC), fimbria (FI)). Quantification of (E) GFAP-positive area and (F) GFAP staining intensity in grey matter areas including CO, HIP and THA.∗p < 0.05, ∗∗p < 0.01, ∗∗∗p < 0.001 for intergroup effects.

Representative pictures of brain cross-sections stained for DCX as a marker for neurogenesis, and IBA-1 as a marker for activated microglia are provided in Fig. 7A. In Aging-Chow and HFD groups (both 8 months of age) neurogenesis was reduced by ∼80 % compared with the Young-Chow mice (3 months old) but no difference was observed between 8-month-old Aging-Chow and 8 month-old HFD mice (Fig. 7B). Compared with the Young-Chow reference group, IBA-1 positive area was increased in Aging-Chow and HFD groups in grey matter (i.e. cortex, hippocampus, and thalamus) and in the corpus callosum (Fig. 7C). Microglial cells were also more numerous in these brain regions in the Aging-Chow and HFD groups (Fig. 7D). No difference in neuroinflammation was observed in the fimbria. The aging-related increases in IBA-1-positive area and IBA-1-positive cells were accompanied by an increase in GFAP-positive area in both Aging-Chow (8 months of age) and HFD (8 months of age) groups compared with the 3-month-old Young-Chow group (Fig. 7E), indicating the development of aging-related astrogliosis. This aging-related astrogliosis was observed in the three grey matter regions investigated (i.e. cortex, hippocampus, thalamus, Fig. 7F). No group differences in GFAP staining intensity were observed in the cortex and thalamus. In the hippocampus however, the intensity was lower in the HFD group compared with the Young-Chow group and similar between the Old-Chow and Young-Chow group.

**Fig. 8:**
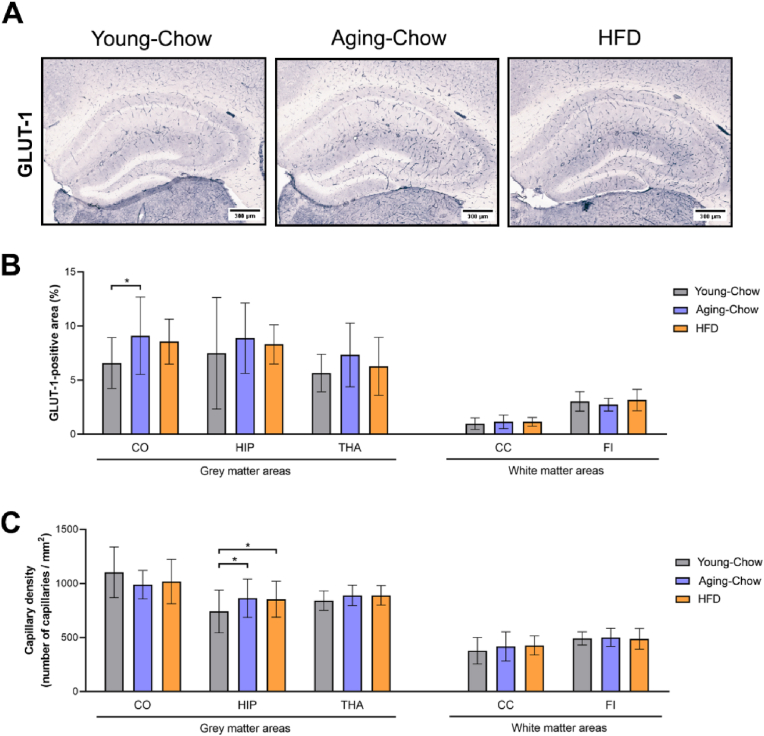
**Cerebrovascular integrity.** (A) Representative pictures of coronal brain cross-sections stained for GLUT-1 as a marker for capillaries or stained with Masson's trichrome to detect atherosclerosis and endothelial dysfunction for Young-Chow (3 months of age), Aging-Chow (8 months of age) and HFD (8 months of age) groups. Quantification of (B) GLUT-1-positive area and (C) capillary density in grey matter areas (i.e. cortex (CO), hippocampus (HIP), thalamus (THA)) and white matter areas (i.e. corpus callosum (CC), fimbria (FI)). ∗p < 0.05, for intergroup effects.

Vascular integrity was assessed using GLUT-1 as a marker for capillaries. Representative pictures of GLUT-1-stained cross-sections are provided in Fig. 8A. Compared with Young-Chow mice, 8-month-old Aging-Chow mice showed higher GLUT-1-positive area in the cortex (Fig. 8B). The capillary density was specifically increased in the hippocampus of both Aging-Chow mice and HFD mice compared with the Young-Chow group (Fig. 8C).

### 3.7 Based on hippocampal gene expression, aging downregulates hippocampal function while HFD feeding activates neuroinflammatory pathways

Hippocampal mRNA transcriptomics datasets were used to identify biological pathways affected by aging on chow diet (Table 1 and Supplementary Table S13) and by HFD feeding (Table 2, Table 3 and Supplementary Table S14). Several canonical pathways related to metabolic and neuronal functions were suppressed during the aging process on chow diet, including ‘G-Protein Coupled Receptor Signaling, ‘CREB Signaling in Neurons and ‘Calcium Signaling’ (Table 1). Furthermore, pathways related to the reorganization of actin filament network (e.g., ‘Signaling by Rho Family GTPases’, ‘Regulation of Actin-based motility by Rho’) were found to be inactivated, and ‘Axonal Guidance Signaling’ was also significantly affected by aging (pathway without direction, hence no z-score available).

**Table 1:**
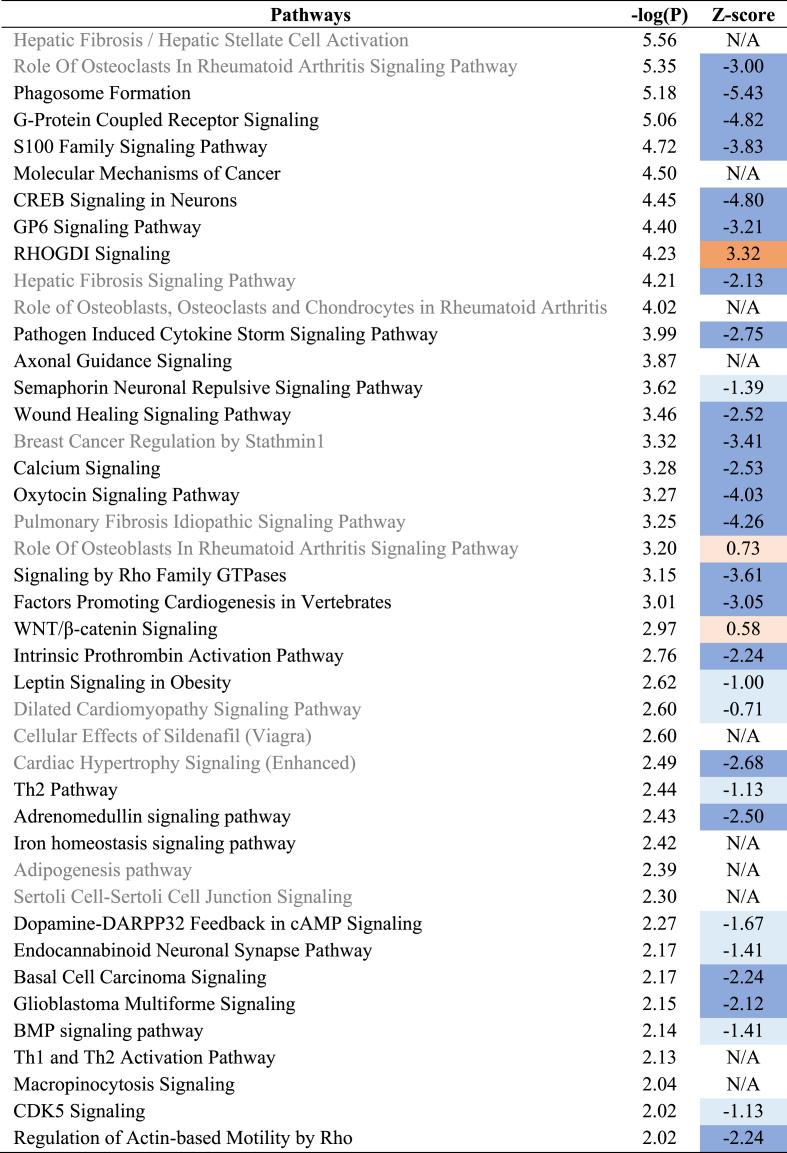
Canonical pathways analysis based on hippocampal gene expression in 8 month-old Aging-Chow group vs 3 month-old Young-Chow group.

Significantly enriched canonical pathways are displayed (p ≤ 0.01 (-log(P-value) ≥ 2)). The Z-score indicates the predicted activation of a canonical pathway: Z-score ≤ −2 indicates relevant inhibition of the pathway (shown in dark blue); Z-score ≥2 indicates relevant activation of the pathway (shown in dark orange).

**Table 2:**
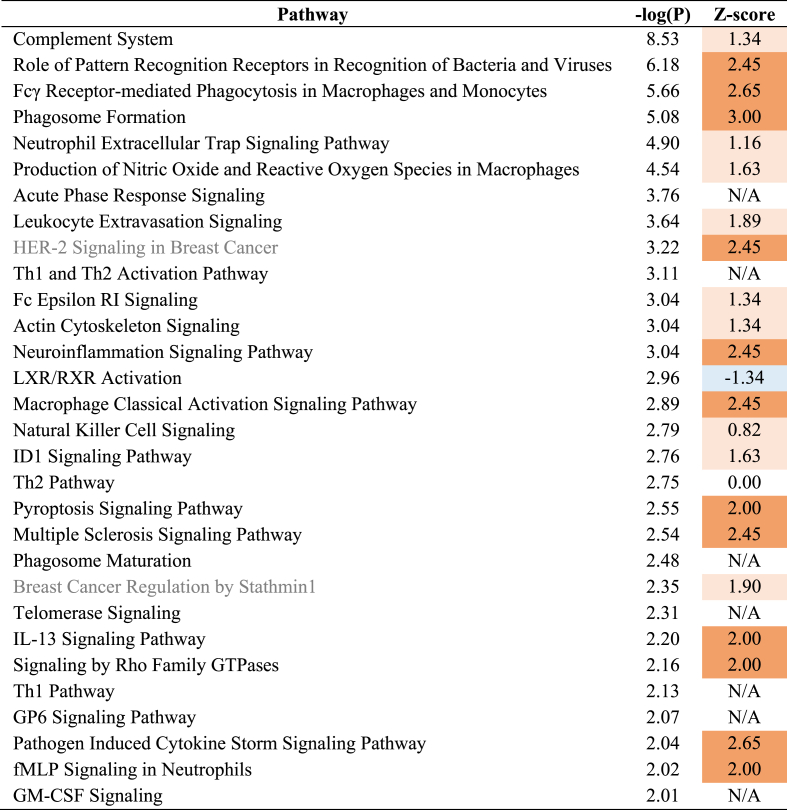
Canonical pathways analysis based on hippocampal gene expression in 8 month-old HFD group vs 8 month-old Aging-Chow group.

Significantly enriched canonical pathways are displayed (p ≤ 0.01 (-log(P-value) ≥ 2)). The Z-score indicates the predicted activation of a canonical pathway: Z-score ≤ −2 indicates relevant inhibition of the pathway (shown in dark blue); Z-score ≥2 indicates relevant activation of the pathway (shown in dark orange).

**Table 3:**
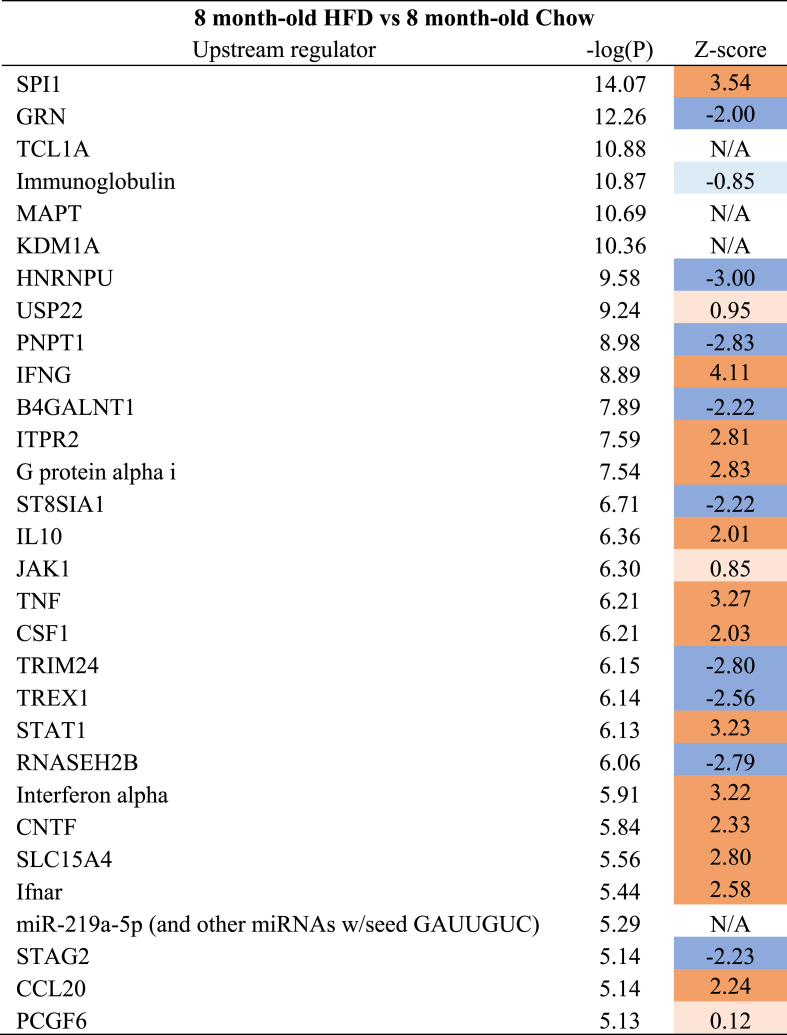
30 most significantly enriched upstream regulator based on hippocampal gene expression in HFD group (8 months old) vs Aging-Chow group (8 months old).

Only 30 most significantly enriched upstream regulators are displayed. The Z-score indicates the predicted regulation of an upstream regulator: Z-score ≤ −2 indicates relevant downregulation of the upstream regulator (shown in dark blue); Z-score ≥2 indicates relevant upregulation of the upstream regulator (shown in dark orange).

At 8 months of age, in comparison with Aging-Chow mice, HFD mice showed directional activation of multiple canonical pathways related to neuroinflammation (e.g., ‘Complement system’, ‘Fcγ Receptor-mediated Phagocytosis in Macrophages and Monocytes’, ‘Neuroinflammation Signaling Pathway’, ‘Macrophage Classical Activation Signaling Pathway’ (Table 2),). The corresponding upstream regulator analysis indicated an increase in hippocampal neuroinflammation in HFD-fed mice compared with chow-fed mice (Table 3): For example, several pro-inflammatory cytokines were activated such as Interferon-ɣ (INFG), Tumor Necrosis Factor (TNF), CCL20.

### 3.8 HFD-fed mice show earlier impairment of memory than chow-fed mice

Short-term memory and spatial learning were first assessed at 3 months of age in the Young-Chow group and at 4 months of age in the Aging-Chow and HFD groups with a MWM test. All groups performed the same and no effects of aging or HFD feeding was yet observed (Fig. S5).

**Fig. 9:**
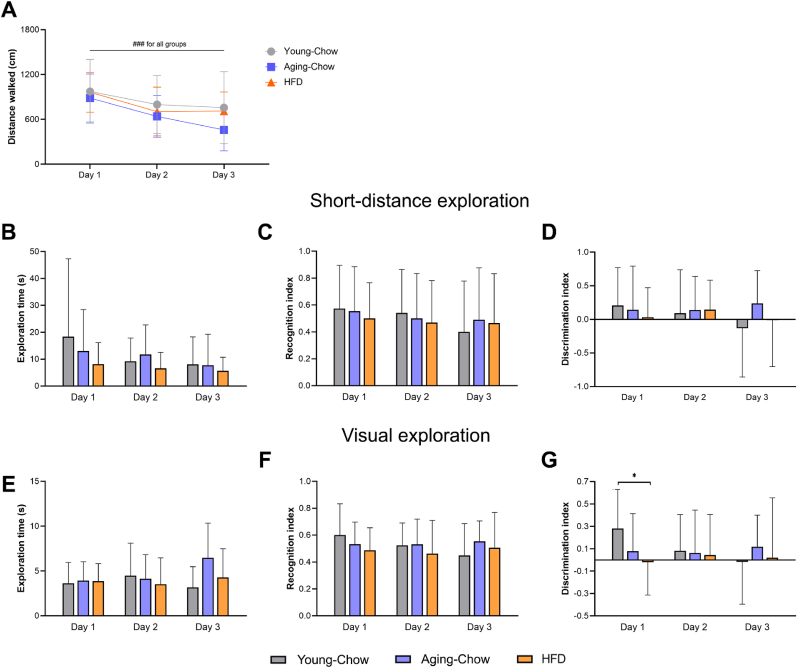
**Object Recognition test (ORT).** An ORT was performed over 3 days in the Young-Chow group (3 months of age), Aging-Chow group (5 months of age) and HFD group (5 months of age) to assess short-term memory and general behavior. (A) The total distance walked was measured over the three ORT days. The (B) total exploration time, (C) recognition index and (D) discrimination index were first determined based on short-distance exploration (nose-point located within 2-cm diameter around the object). In parallel, the (E) total exploration time, (F) recognition index and (G) discrimination index were also determined based on visual exploration (head directed towards the object). Data are shown as mean ± SD. ###p < 0.001 for intragroup effects over time; ∗p < 0.05 for intergroup effects.

Cognitive performance was then evaluated using an ORT at 3 months old in the Young-Chow group and at 5 months old in Aging-Chow and HFD groups. Over the three days of ORT, distance walked by the animals decreased in all the groups (Fig. 9A). The total time during which the mice explored the objects (including familiar and novel objects) were similar in all the groups for both short-distance exploration and visual exploration (Fig. 9, Fig. 8E). There were no group differences in recognition or discrimination indices when the exploration considered only touch and smell of the objects (i.e. short-distance exploration; nose-point located within 2-cm diameter around the objects, Fig. 9C and D). When additionally including visual inspection in the exploration (i.e. head directed towards the object), all the groups spent the same amount of time exploring the novel object (Fig. 9F). However, on day 1 (30-min interval between the familiarization and test phase), the 5-month-old HFD mice had a discrimination index that was slightly negative indicating slightly longer exploration for the familiar object while Aging-Chow mice of the same age showed a positive value close to 0 indicating no preference for either object or a slight preference for the novel object (Fig. 9G). The Young-Chow mice had on average a discrimination index of indicating longer exploration of the novel object and better discrimination between the objects. No difference in discrimination index between the groups was observed on day 2 and 3 of the ORT.

**Fig. 10:**
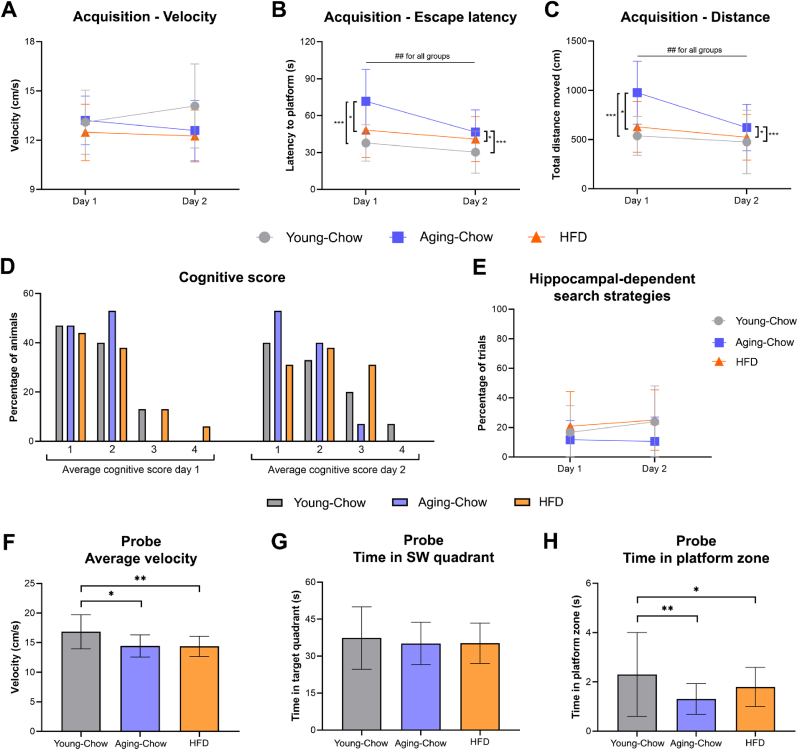
**Reverse Morris Water Maze (rMWM) test.** A rMWM test was performed in the Young-Chow group (3 months of age), Aging-Chow group (8 months of age) and HFD group (8 months of age) to assess short-term memory and spatial learning. (A) Average velocity, (B) escape latency and (C) total distance moved were determined during the two-day acquisition phase for a new platform location (learning phase). (D) Average cognitive score per day (0: thigmotaxis, 1: random search, 2: scanning, 3: chaining, 4: indirect search or semi-focal search or focal search, 5: directed search, 6: direct path). (E) Percentage of hippocampal-search strategies used during the four days of the acquisition phase (scores 4–6 were considered hippocampus-dependent search strategies). During the probe phase, (F) the velocity, (G) the time spent in the South-West (SW) quadrant and (H) the time spent in the platform zone were determined. Data are shown as mean ± SD. ##p < 0.01 for intragroup effects over time; ∗p < 0.05, ∗∗p < 0.01, ∗∗∗p < 0.001 for intergroup effects.

Finally, at 3 months of age of the Young-Chow groups and at 8 months of age for the Aging-Chow group and HFD group, short-term memory and spatial learning were again assessed with a rMWM. During the acquisition phase (learning phase for a new platform location), all mice showed the same velocity (Fig. 10A). The time to find the platform (escape latency) and the distance swam before finding the platform decreased over the two days of the acquisition for all the groups (Fig. 10B and C), indicating that the mice successfully learned the new platform location. The Aging-Chow group (8 months old) showed an overall higher escape latency and a longer distance swam to find the platform, especially on the first day of the acquisition phase. To further characterize search strategies during the learning (acquisition) phase, cognitive scores were allocated based on the search pattern. All the mice showed the same cognitive score and used the same amount of hippocampus-dependent search strategies (Fig. 10D and E). During the probe phase, Aging-Chow and HFD mice (8 months of age) moved significantly slower than the Young-Chow mice (3 months of age, Fig. 10F). After an adjustment for velocity, all the groups spent the same amount of time in the quadrant where the platform was located (Fig. 10G). Both 8-month-old Aging-Chow and HFD mice spent however less time in the platform zone than Young-Chow mice (Fig. 10H).

### 3.9 Correlations between cognition and brain structure and function

To gain further insight into the structural and functional alterations that may underlie age- and HFD feeding-related cognitive decline, associations were analyzed between cognitive performance and parameters related to brain structure and function (Supplementary Table S15). Average cortical thickness was negatively associated with the discrimination index in the second day of the ORT, while hippocampal volume was not associated with any brain parameter. Higher fractional anisotropy in white matter, especially in the optic tract, but also in somatosensory cortex and basal ganglia (i.e., caudate, putamen, and globus pallidus) was associated with better performance in the ORT.

Higher CBF in the cortex and hippocampus under normal gas conditions was associated with better performance in the reverse MWM probe. On the contrary, higher CBF in the thalamus was associated with poorer performance in the ORT. In addition, higher CBF under vasoconstrictive conditions (i.e., under pure oxygen) in the cortex and thalamus was related to poorer recognition of the novel object in the ORT. Consistent with this, lower cerebral vasoreactivity (i.e., higher values) was associated with poorer recognition of the novel object.

In addition, higher rs-FC between the hippocampus and auditory cortex was associated with better performance in the MWM, while higher rs-FC between the hippocampus and motor cortex was associated with better performance in the ORT.

Finally, based on (immuno)histopathological analyses of the brains, GLUT-1-positive area in the cortex and the capillary density in the hippocampus were negatively correlated with performance in the reverse MWM.

## 4 Discussion

Using brain MRI, cognitive tests and histopathological analyses, the present study investigated how obesity and normal aging interact in the alteration of brain structure and function during mid-adulthood. This study demonstrated that the Ldlr-/-.Leiden mouse model replicates early signs of aging-related brain changes: On chow diet, these mice exhibited age-related decreases in cortical thickness, cerebral perfusion and cortico-hippocampal connectivity, in addition to the development of neuroinflammation, cerebrovascular remodeling and reduction in neurogenesis. Based on hippocampal gene expression analyses, incipient aging in this mouse model particularly suppressed processes related to metabolic and neuronal functions. Importantly, these structural and functional alterations seemed to appear few months before cognitive defects could be noticed. Upon HFD feeding, Ldlr-/-.Leiden mice exhibited similar features but the decline in memory function started at a younger age. In addition, HFD-fed mice exhibited increases in CBF and impaired white matter tract integrity, potentially contributing to the faster cognitive decline. Hippocampal gene expression analyses revealed that HFD feeding activates multiple neuroinflammatory pathways, which may also underlie HFD-induced accelerated cognitive decline.

Decline in cortical thickness is an established feature of normal aging and has been linked to poorer executive functioning (Burzynska et al., 2012; Thambisetty et al., 2010). While obesity was shown to also induce cortical thinning and atrophy in humans (Shaw et al., 2018) and in mice (Patel et al., 2024), we did not find differences in age-related cortical thinning between HFD-fed mice and chow-fed mice. This is consistent with other studies in this mouse model that also showed no effect of HFD feeding on global cortical thickness (Arnoldussen et al., 2022). Further research is needed to determine whether these discrepancies with other studies are due to the Ldlr-/-.Leiden strain itself or due to the type and duration of the HFD feeding.

In addition to cortical atrophy, Ldlr-/-.Leiden mice exhibited progressive alterations in white matter integrity as observed with FA changes in DTI. Higher FA values are considered to reflect proper fiber density and adequate myelination in white matter tracts, while lower FA values may rather indicate impairments (Mori and Zhang, 2006). During aging processes in humans, changes in FA have been reported and three phases were described: typically, FA first increases (up to ∼33 years of age), then appears to be stable (second phase), and finally decreases in the last phase (from ∼65 years onwards) (Westlye et al., 2010). These age cut-offs provide a rough indication of the dynamics and FA-maxima may differ between white matter regions (Westlye et al., 2010; Behler et al., 2021). In the present study, FA increased with age in the optic tract of both chow-fed and HFD-fed mice, indicating that fiber density and myelination have not yet reached their maximum. This suggests ongoing maturation of white matter tracts, which is consistent with other studies showing continued myelination and neuronal growth in young adult mice, leading to increased FA values (Piekarski et al., 2023; Yon et al., 2020). In the corpus callosum and fornix, FA remained stable during aging, suggesting that fiber density and myelination in these regions are already in the second phase. However, these results should be interpreted with caution, as tissue loss or damage may also lead to increased or unchanged FA due to the diversity of fiber populations with different trajectories and the presence of crossing fibers (Figley et al., 2022). In parallel, in all white matter regions investigated by us, FA significantly decreased due to HFD feeding as early as 5 months of age. These findings are consistent with human studies showing that FA is decreased in individuals with obesity compared with lean and overweight people (Stanek et al., 2011). Spectroscopic studies further supported that the deterioration of white matter tracts may be accelerated in persons with high BMI (Gazdzinski et al., 2008). Our findings corroborate the hypothesis of a faster degradation of white matter microstructure upon HFD feeding, which is consistent with theories suggesting that brain aging is accelerated by obesity (Ronan et al., 2016).

Besides structural alterations, CBF impairment is also known as an established key feature of normal aging and it has been linked to cognitive decline and increased risk of dementia (Wolters et al., 2017; Martin et al., 1991). Consistently, in the present study, Ldlr-/-.Leiden mice exhibited a gradual decrease in grey matter CBF during normal aging. Maybe unexpectedly, the aging mice on HFD exhibited a higher CBF than the normal aging mice on chow diet. While some studies showed CBF decline in patients with obesity (Amen et al., 2020), more refined studies in patients with mild cognitive impairment revealed that CBF may first increase during the early phase of cognitive impairment and then decrease in more advanced stages (Thomas et al., 2021). The latter study suggests that compensatory mechanisms are part of an adaptive response of the body to ensure appropriate perfusion of brain tissue in the early stages of neurodegenerative disorders. In the present study, it is therefore possible that the aging-related decrease in CBF observed in the chow-fed group was masked by a compensatory increase in CBF in HFD-fed mice, resulting in a seemingly stable CBF upon HFD feeding. The hypothesis that increases in CBF can be detrimental is further supported by the negative correlations observed in this study between thalamic CBF and cognitive performance in the ORT. This increase may, for instance, compensate for the white matter tract disruption observed upon HFD feeding. However, previous studies in Ldlr-/-.Leiden mice have also shown decreased or unaffected CBF on HFD feeding (Arnoldussen et al., 2017, 2022; Tengeler et al., 2020). Given that animal studies investigating longitudinal changes in cerebral perfusion are still scarce, more research is needed to delineate the different temporal phases of CBF impairment in lean and obese mice.

In this study, we also observed a global decrease in brain rs-FC during aging in both chow-fed and HFD-fed mice. This age-related decline was particularly prominent between the hippocampus and cortical regions. These results are consistent with previous studies in humans showing that aging particularly impairs connectivity at rest in the default-mode network, which involves the hippocampus (Ferreira and Busatto, 2013). Salami et al. further described an age-related reduction in cortico-hippocampal rs-FC, along with an increase of intrahippocampal rs-FC, which correlated with memory impairment (Salami et al., 2014). Consistent with this, upon HFD feeding, we observed an age-related increase in rs-FC between right ventral hippocampus and left dorsal hippocampus, which was not observed in aging chow-fed mice. However, it is important to note that there were no differences between older chow-fed and HFD-fed mice with the 3-month old young control group, suggesting that the effect of normal aging and HFD feeding is rather mild.

Histological analyses showed that Ldlr-/-.Leiden mice developed aged-related astrogliosis, which was predominant in the hippocampus. In the hippocampus, we further observed an age-related cerebrovascular remodeling since capillary density (based on GLUT-1 staining) increased during aging. While aging has been associated with decreased cerebral vascular density and length (Zhang et al., 2023), angiogenesis may also occur as an adaptive response to hypoxia but the capacity to mount such responses may decline at later age (Brown and Thore, 2011). In parallel, we found that aged Ldlr-/-.Leiden mice (at 8 months of age) on chow diet exhibit significant neuroinflammation (including microgliosis and astrogliosis) in grey matter and decreased hippocampal neurogenesis compared with young mice (3 months old). Altogether, these results indicate that the Ldlr-/-.Leiden mouse model reflects age-related neuroinflammation and vascular remodeling with a specific sensitivity of the hippocampus to aging. The hippocampus, as the main brain region involved in memory function, presents a high level of neuroplasticity and is reportedly particularly vulnerable to deleterious alterations related to aging and neurodegeneration (Bartsch and Wulff, 2015). In line with this, we found that grey matter FA was particularly decreased in the hippocampus during aging, and HFD-induced increase in CBF was first noticed in the hippocampus after 12 weeks of HFD feeding but only after 27 weeks in the cortex and thalamus. It is however important to note that hippocampal volume itself was not yet affected by age or HFD-feeding. Although negative relationships between hippocampal volumetry and memory performance are well established in neurogenerative disease such as Alzheimer's disease, the existence of such negative relationships in healthy older adults are still controversial (Van Petten, 2004).

Under the experimental conditions used herein, we observed that there may be an interaction between age and HFD, which should be taken into account when comparing studies or interpreting the effect of HFD feeding on brain structure and function. Based on previous reports, it seems that, depending on the age of the mice, the duration of HFD feeding, and the brain region investigated, IBA-1 expression can be differently affected (Seidel et al., 2023; Arnoldussen et al., 2017, 2022; Tengeler et al., 2020). This could, for instance, be explained by an immunophenotypic shift of microglia toward a phagocytic activity state characterized by the expression of other markers like CD68 (Seidel et al., 2023). Although in the present study we did not observe an histological effect of HFD feeding on IBA-1 or GFAP, a genome wide gene expression analysis of the hippocampus revealed multiple neuroinflammatory pathways and upstream regulators being significantly upregulated in 8 month-old HFD-fed mice compared with chow-fed mice of the same age, advocating the analysis of other markers of neuroinflammation in future studies. Furthermore, the interaction between age and HFD may also impact the effect of HFD feeding on functional alterations in the brain: For example, at 4 months of age, HFD-fed mice exhibited lower rs-FC than chow-fed mice. While rs-FC in HFD-fed mice remained at lower values, rs-FC strongly decreased in chow-fed mice between 4 and 5 months of age. As a consequence, after 5 months of age, no differences between the groups were detectable anymore. This illustrates that it is important to carefully consider the age of the mice at the start of a study, and select appropriate time frames when examining obesity-induced brain alterations. In addition, despite examining for statistical outliers, marked biological variations were occasionally observed among the mice. Future studies that focus on specific subgroups of animals (such as those categorized by their response to HFD feeding), could enhance our understanding of how obesity and metabolic disturbances affect brain function.

In contrast with the alterations in brain structure and function (which occurred relatively early), cognitive impairments in Ldlr-/-.Leiden mice on a chow diet were observed much later, namely at 8 months of age (i.e., decreased short-term (spatial) memory in the rMWM test). Explorative correlations analyses revealed that performance in the reverse MWM is positively associated with CBF in the cortex and hippocampus, but negatively associated with GLUT-1-positive area in the cortex and capillary density in the hippocampus. Since chow-fed mice exhibited age-related decreases in CBF and increased hippocampal capillary density, these associations suggest that, in normal aging (on chow diet), decreases in CBF and cerebrovascular remodeling, particularly in the hippocampus, may contribute to memory decline.

In the context of HFD-feeding, memory impairment was already noticed at 5 months of age, thus 3 months earlier compared with chow feeding, because HFD-fed mice were not able to discriminate the familiar and novel objects in the ORT. In addition, HFD-fed mice showed impaired short-term memory performance in the rMWM at 8 months of age. These observations indicate that short-term memory is impaired at a younger age in HFD-fed mice than in chow-fed mice, suggesting an acceleration of cognitive decline in obesity conditions. Correlation analyses showed that lower FA in white matter, especially in the optic tract, but also in somatosensory cortex and basal ganglia, is associated with poorer performance in the ORT. They also revealed that higher CBF and impaired vasoreactivity in the thalamus, as well as decreased rs-FC between the hippocampus and motor cortex, are associated with poorer performance in the ORT. Given that HFD-fed mice exhibited increases in thalamic CBF, impaired vasoreactivity and decreased FA compared with age-matched Chow controls, these correlations support the view that, under HFD feeding, faster cognitive decline may be mediated by the degradation of white matter microstructure and disturbances in brain blood perfusion.

To conclude, this study demonstrates that the Ldlr-/-.Leiden mouse model exhibits crucial features of age-related neurodegeneration, including cortical thinning, deficits in cerebral blood perfusion and decreased brain connectivity, all of which may contribute to the observed age-related cognitive decline on chow diet. In this mouse model, HFD-induced obesity accelerates the development of memory deficits, which may be partly mediated by the impairment of white matter tract integrity and obesity-associated disturbances in brain perfusion. Hippocampal gene expression analyses demonstrated an activation of neuroinflammatory processes in comparison with chow diet, which may additionally underlie these obesity-induced functional alterations in the brain. This mouse model therefore appears to adequately replicate key processes of human pathology, providing a valuable tool for the investigation of potential future pharmacological or dietary interventions to alleviate brain dysfunction in the context of aging and obesity.

## CRediT authorship contribution statement

**Florine Seidel:** Writing – review & editing, Writing – original draft, Visualization, Investigation, Formal analysis, Data curation, Conceptualization. **Martine C. Morrison:** Writing – review & editing, Writing – original draft, Supervision, Project administration, Funding acquisition, Conceptualization. **Ilse Arnoldussen:** Writing – review & editing, Writing – original draft, Supervision, Conceptualization. **Vivienne Verweij:** Writing – review & editing, Writing – original draft, Investigation. **Joline Attema:** Writing – review & editing, Writing – original draft, Investigation. **Christa de Ruiter:** Writing – review & editing, Writing – original draft, Investigation. **Wim van Duyvenvoorde:** Writing – review & editing, Writing – original draft, Investigation. **Jessica Snabel:** Writing – review & editing, Writing – original draft, Investigation. **Bram Geenen:** Writing – review & editing, Writing – original draft, Investigation. **Ayla Franco:** Writing – review & editing, Writing – original draft, Investigation. **Maximilian Wiesmann:** Writing – review & editing, Writing – original draft, Supervision, Project administration, Formal analysis, Conceptualization. **Robert Kleemann:** Writing – review & editing, Writing – original draft, Supervision, Project administration, Funding acquisition, Conceptualization. **Amanda J. Kiliaan:** Writing – review & editing, Writing – original draft, Supervision, Project administration, Conceptualization.

## Data statement

The datasets included in this study are available from the corresponding author upon request. Hippocampal RNAseq data are available in the Gene Expression Omnibus (GEO) repository (https://www.ncbi.nlm.nih.gov/gds) under the accession number GSE294054.

## Funding source

The study was supported by internal research programs of 10.13039/501100019926TNO “PMC Brain Health”, “ERP Body brain interactions” and “ERP Brain Power”.

## Declaration of competing interest

The authors declare that they have no known competing financial interests or personal relationships that could have appeared to influence the work reported in this paper.

## Data Availability

Data will be made available on request.
