# Supplementary material for: Obesity accelerates age-related memory deficits and alters white matter tract integrity in Ldlr-/-.Leiden mice
Source: Brain Behav Immun Health. 2025 Apr 15;45:100991. doi: 10.1016/j.bbih.2025.100991 (PMC12032874; doi:10.1016/j.bbih.2025.100991)
Supplement: Multimedia component 1 [file mmc1.docx]

# Supplementary material

## Supplementary methods

1. Brain MRI

Brain MRI was performed using a 11.7T BioSpec Avance III small animal MR system (Bruker Biospin, Ettlingen, Germany) with Paravision 6.0.1 software (Bruker) as already extensively described [1–3]. Prior to MRI, mice were anesthetized with isoflurane (3.5% for induction, 1.8% for maintenance) in a 1:2 oxygen-medical air mixture. The head of the mice was placed in a stereotactic holder to limit motion and eye ointment was placed on the eye to prevent dehydration. Both the respiration rate (pneumatic cushion) and body temperature (rectal probe) were monitored with a monitoring system (Small Animal Instruments Inc, Stony Brook, NY, USA).

1.1 Hippocampal volume and cortical thickness

Cortical thickness and hippocampal volume were measured in T2-weighted coronal images using ImageJ (v1.53, National Institutes of Health, United States) and the mouse brain atlas of Franklin and Paxinos [4]. Thickness of the auditory cortex (AUC, bregma -2.46), motor cortex (MC, bregma 1.10), somatosensory cortex (SSC, bregma -0.94) and visual cortex (VC, bregma -2.46) were measured manually. For each region, the measures from left and right hemispheres were averaged. Hippocampus volume was manually measured on 6 consecutive slices to cover the entire hippocampus (bregma -0.94 to -3.40). The measurements of the 6 slices from both hemisphere were summed and multiplied by slice thickness (0.5 mm). The overall cortical thickness was finally calculated as the average of AUC, MC, SSC and VC cortical thickness.

1.2 White matter and grey matter integrity

Diffusion Tensor Imaging (DTI) was performed to assess grey and white matter integrity based on fractional anisotropy and mean diffusivity: fractional anisotropy in white matter provides indication on the degree of myelination and fiber density, whereas mean diffusivity in grey matter describes an inverse measure of membrane density [5]. These two parameters were measured in white matter areas (corpus callosum, fornix and optic tract) and grey matter areas (AUC, MC, SSC, VC, hippocampus and combined caudate nucleus, globus pallidus, putamen). Measures for left and right hemispheres were averaged for each region and were eventually expressed as the average fractional anisotropy and the average mean diffusivity in grey matter and white matter.

1.3 Cerebral blood flow (CBF)

CBF was assessed using an Arterial Spin Labeling sequence with flow-sensitive alternating inversion recovery (FAIR) method [6]. Arterial Spin Labeling was first performed under normal gas mix (1:2 oxygen - medical air). The Arterial Spin Labeling sequence was repeated after switching to pure oxygen to induce vasoconstriction. Cerebral vasoreactivity, defined as the ability of the cerebrovasculature to adapt to constrict or dilate, was calculated by subtracting the CBF measured under vasoconstrictive conditions from the CBF measured under normal gas mix, divided by the CBF measured under normal gas mix [6]. CBF under normal gas mix, CBF under vasoconstrictive conditions and vasoreactivity were measured in the cortex, hippocampus, and thalamus.

1.4 Functional connectivity

Resting-state functional MRI acquisition was used to assess functional connectivity between specific regions of interest implicated in multiple cognitive and motor processes: ventral and dorsal hippocampus, auditory, somatosensory, motor, and visual cortical regions. Functional connectivity was determined from blood oxygen-dependent (BOLD) time series using total correlation analyses [7]. Z-scores were obtained after R-to-Z transformations and used for statistical analysis.

## Supplementary figures


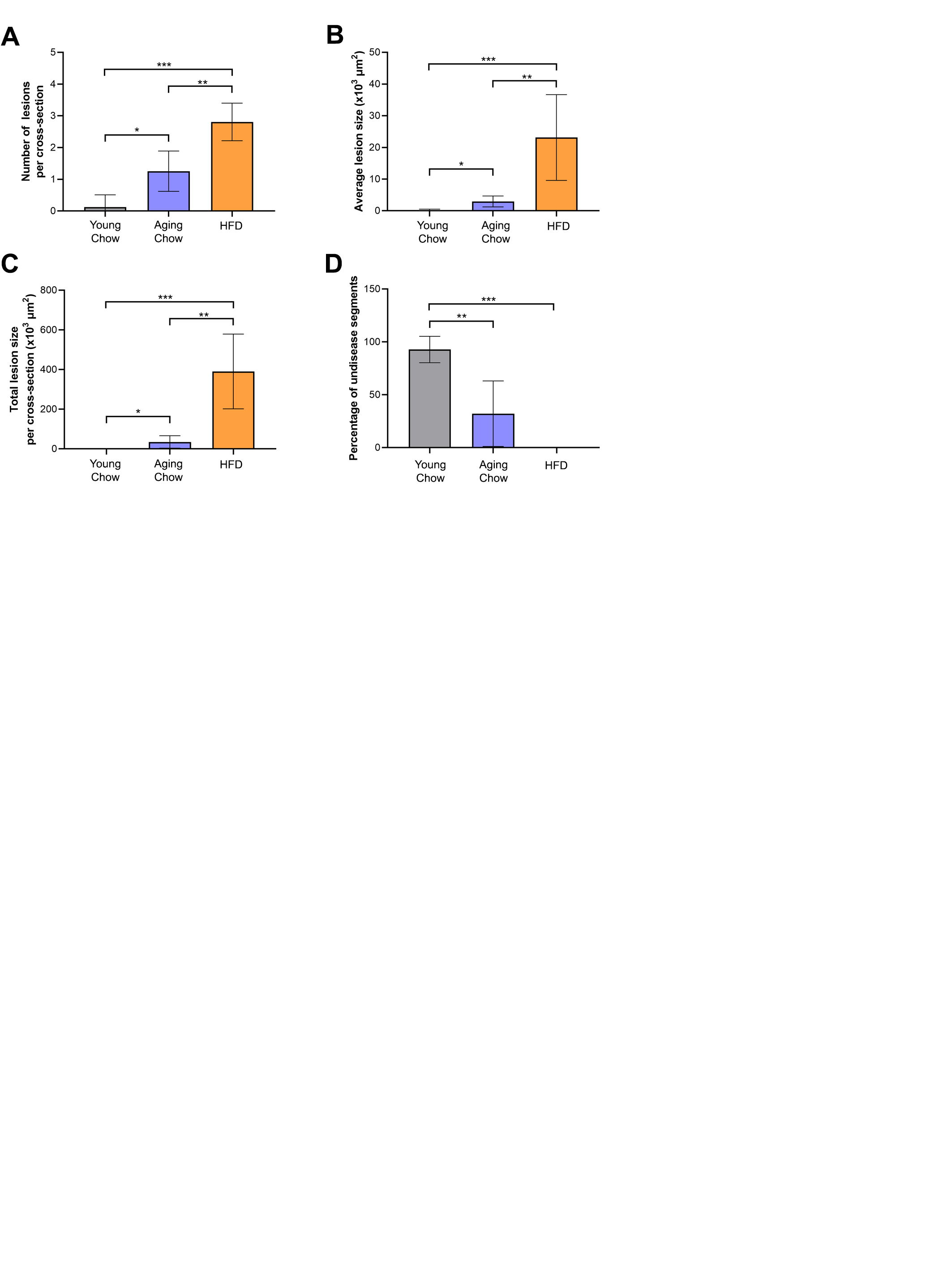


**Supplementary figure S1: Atherosclerosis.** (A) Number of atherosclerotic lesions per cross-section, (B) average lesion size, (C) total lesion size per cross-section and (D) percentage of undiseased segments were analysed in cross-sections of the aortic roots stained with hematoxylin-phloxine-saffron for Young-Chow (3 months of age), Aging-Chow (8 months of age) and HFD (8 months of age) groups. Data are shown as mean ± SD. * p<0.05, ** p<0.01, *** p<0.001 for intergroup effects.


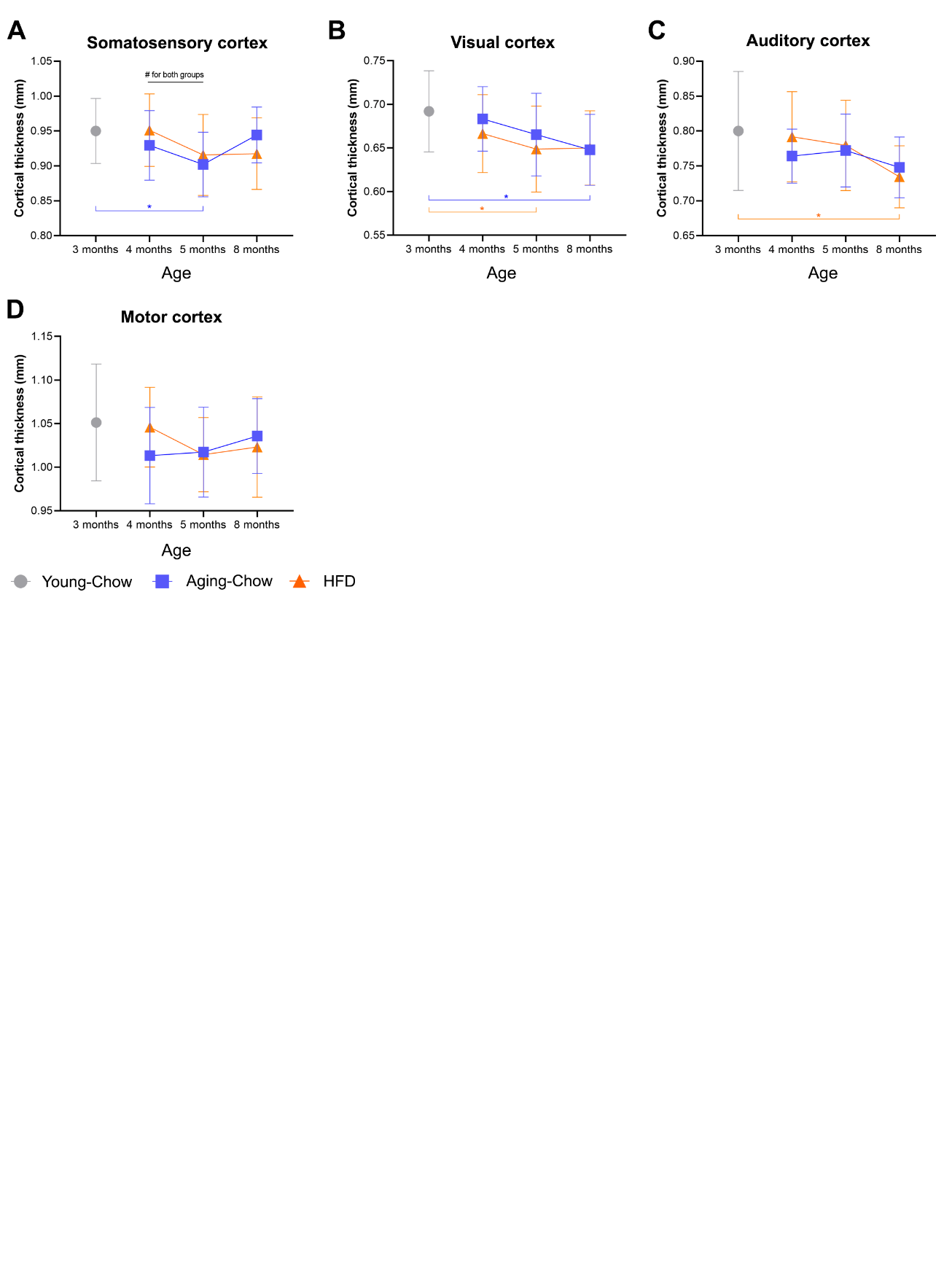


**Supplementary figure S2: Cortical thickness.** Thickness of (A) somatosensory cortex, (B) visual cortex, (C) auditory cortex, and (D) motor cortex. Data are shown as mean ± SD. # p<0.05 for intragroup effects over time; * p<0.05 for intergroup effects.

**
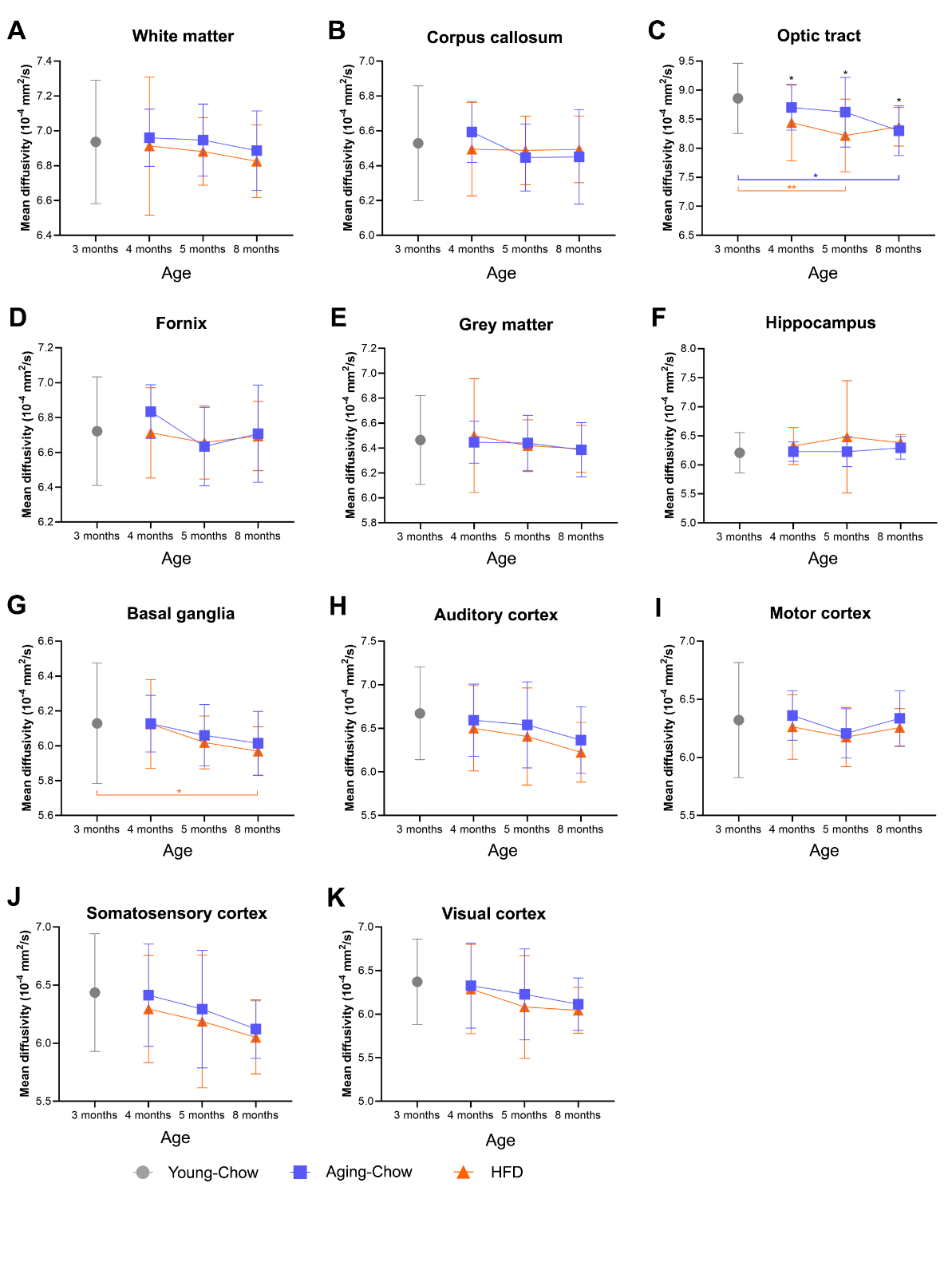
**

**Supplementary figure S3: Mean diffusivity in grey and white matter based on DTI.** Using DTI, mean diffusivity was measured in (A) white matter including the (B) corpus callosum, (C) optic tract and (D) fornix, and in (E) grey matter including the (F) hippocampus, (G) basal ganglia (caudate nucleus, putamen and globus pallidus), (H) auditory cortex, (I) motor cortex, (J) somatosensory cortex and (K) visual cortex. * p<0.05, ** p<0.01 for intergroup effects.


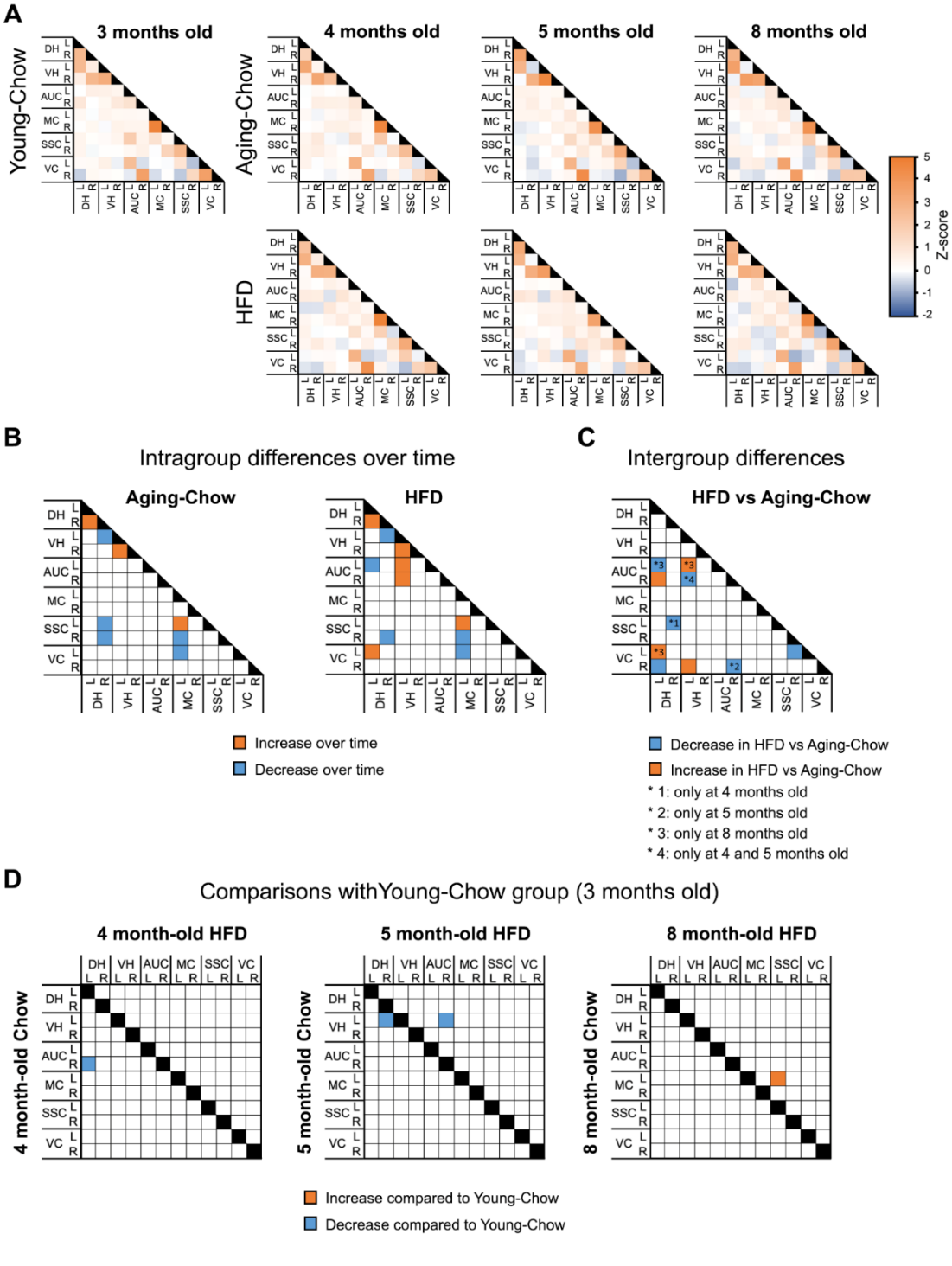


**Supplemental figure S4: Resting-state functional connectivity based on partial correlations.** (A) Heatmaps showing resting-state functional connectivity (rs-FC, partial correlations) between brain regions in Young-Chow (3 months old), Aging-Chow and HFD groups. (B) Overall intragroup changes in rs-FC between 4 and 8 months old in Aging-Chow and HFD groups. (C) Group differences in rs-FC between HFD and Aging-Chow groups. (D) Comparison of rs-FC of 4 month-old, 5 month-old and 8-month old Aging-Chow and HFD groups with rs-FC of the Young-Chow reference group (3 months old). Abbreviations: (DH) dorsal hippocampus; (VH) ventral hippocampus; (AUC) auditory cortex; (MC) motor cortex; (SSC) somatosensory cortex; (VC) visual cortex in left (L) and right (R) hemispheres.

**
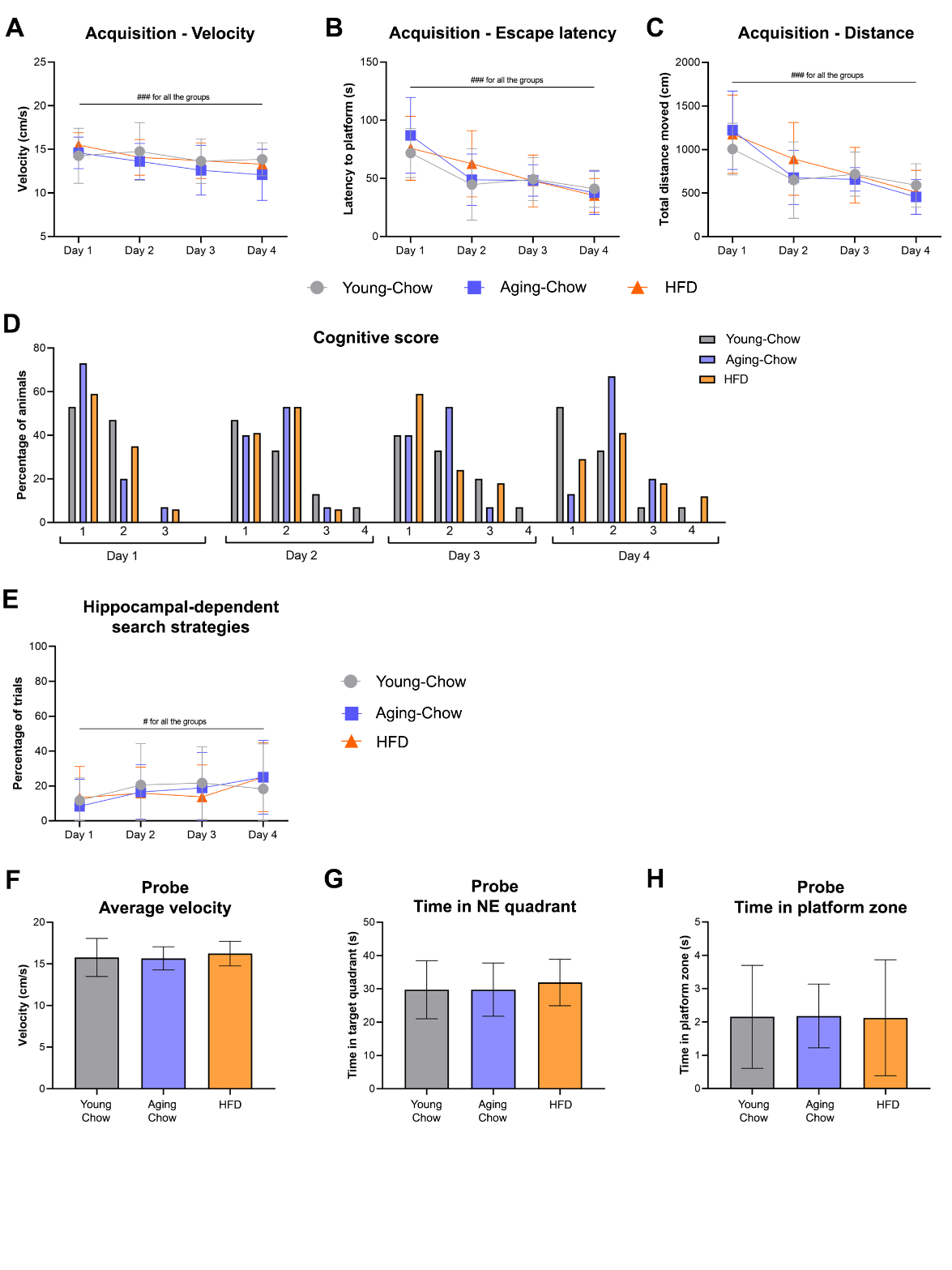
**

**Supplementary figure S5: Morris Water Maze test.** A Morris Water Maze test was performed in the Young-Chow group (3 months old), the Aging-Chow group (4 months old) and the HFD group (4 months old) to assess short-term memory and spatial learning. (A) Average velocity, (B) escape latency and (C) total distance moved were monitored during the four-day acquisition phase (learning phase). (D) Average cognitive score per day (0: thigmotaxis, 1: random search, 2: scanning, 3: chaining, 4: indirect search or semi-focal search or focal search, 5: directed search, 6: direct path).(E) Percentage of hippocampal-search strategies used during the four days of the acquisition phase (scores 4-6 were considered hippocampus-dependent search strategies). During the probe phase, (F) the velocity, (G) the time spent in the North-East (NE) quadrant and (H) the time spent in the platform zone were determined. Data are shown as mean ± SD. # p<0.05, ### p<0.001 for intragroup effects over time.

## Supplementary tables

**Supplemental Table S1: First antibodies.**

**
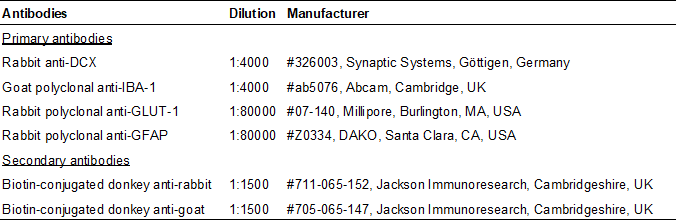
**

Abbreviations: (DCX) doublecortin; (GFAP) glial fibrillary acidic protein; (GLUT-1) glucose transporter 1 ; (IBA-1) ionized calcium-binding adapter molecule 1.

**Supplemental Table S2: Summary statistics of in vivo general parameters.**

**Supplemental Table S3: Summary statistics of atherosclerosis analysis for Young-Chow (3 months of age), Aging-Chow (8 months of age) and HFD (8 months of age) groups.**

**Supplemental Table S4: Summary statistics of cortical thickness and hippocampus size.**

**Supplemental Table S5: Summary statistics of white and grey matter integrity (DTI).**

**Supplemental Table S6: Summary statistics of CBF and vasoreactivity (Arterial Spin Labeling).**

**Supplemental Table S7: Summary statistics of rs-FC based on total correlations.**

Abbreviations: (DH) dorsal hippocampus; (VH) ventral hippocampus; (AUC) auditory cortex; (MC) motor cortex;SSC) somatosensory cortex; (VC) visual cortex in left (L) and right (R) hemispheres.

**Supplemental Table S8: Summary statistics of rs-FC based on partial correlations.**

Abbreviations: (DH) dorsal hippocampus; (VH) ventral hippocampus; (AUC) auditory cortex; (MC) motor cortex; (SSC) somatosensory cortex; (VC) visual cortex in left (L) and right (R) hemispheres.

**Supplemental Table S9: Summary statistics of brain histology for Young-Chow (3 months of age), Aging-Chow (8 months of age) and HFD (8 months of age) groups.**

Abbreviations: (DCX) doublecortin; (GFAP) glial fibrillary acidic protein; (GLUT-1) glucose transporter 1; (IBA-1) ionized calcium-binding adapter molecule 1.

**Supplemental Table S10: Summary statistics of MWM test for Young-Chow (3 months of age), Aging-Chow (4 months of age) and HFD (4 months of age) groups.**

**Supplemental Table S11: Summary statistics of ORT for Young-Chow (3 months of age), Aging-Chow (5 months of age) and HFD (5 months of age) groups.**

**Supplemental Table S12: Summary statistics of reverse MWM for Young-Chow (3 months of age), Aging-Chow (8 months of age) and HFD (8 months of age) groups.**

**Supplementary table S13: Upstream regulator analysis based on hippocampal gene expression in Aging-Chow group (8 months old) vs Young-Chow group (3 months old).**

| **8 month-old Chow vs 3 month-old Chow** | | |
| --- | --- | --- |
| Upstream regulator | -log(P) | Z-score |
| TNF | 11.37 | -2.10 |
| STAT3 | 8.96 | -2.20 |
| ESR1 | 8.65 | -3.71 |
| TP53 | 7.98 | -2.09 |
| CREB1 | 7.91 | -0.10 |
| AGT | 7.38 | -4.08 |
| PGR | 7.26 | 1.64 |
| TGFB1 | 7.16 | -4.14 |
| DMD | 7.12 | -0.15 |
| SORL1 | 7.07 | -3.71 |
| ZNF503 | 7.03 | 0.31 |
| TAFAZZIN | 6.89 | -0.56 |
| ESR2 | 6.30 | -2.38 |
| SP1 | 6.09 | -2.12 |
| HTT | 6.00 | 0.56 |
| BMP7 | 5.98 | -0.66 |
| IGF1 | 5.96 | -1.62 |
| RPA1 | 5.87 | N/A |
| IFNG | 5.80 | 0.15 |
| FFAR3 | 5.74 | 0.90 |
| MRTFB | 5.59 | -2.62 |
| TEAD2 | 5.55 | -2.33 |
| MAP2K1 | 5.52 | -1.59 |
| ARHGAP5 | 5.52 | -2.22 |
| FOXO1 | 5.51 | -2.43 |
| TEAD3 | 5.47 | -1.94 |
| OTX2 | 5.39 | -3.16 |
| FOXM1 | 5.36 | -2.54 |
| MYOCD | 5.29 | -1.21 |
| IL1B | 5.28 | 0.38 |
| SMARCA4 | 5.28 | -2.34 |
| TEAD4 | 5.26 | -1.66 |
| KDM1A | 5.23 | 1.09 |
| CG | 5.22 | -0.68 |
| WNT3A | 5.21 | -3.21 |
| BIRC5 | 5.17 | N/A |
| Vegf | 5.10 | -3.01 |
| IKBKB | 5.07 | -3.31 |
| SMARCA5 | 5.00 | -0.37 |
| ZEB1 | 4.94 | -2.11 |
| MYOD1 | 4.91 | -3.42 |
| IL4 | 4.90 | -3.28 |
| PDGF BB | 4.87 | -1.83 |
| TCF7L2 | 4.80 | -1.68 |
| TFRC | 4.77 | 0.63 |
| KLF5 | 4.66 | -1.09 |
| ID2 | 4.63 | 2.33 |
| GRIN1 | 4.57 | 0.63 |
| TWIST1 | 4.57 | -1.96 |
| PRKAA2 | 4.57 | 0.11 |
| IL13 | 4.57 | -2.05 |
| ERBB2 | 4.57 | -1.82 |
| FRS2 | 4.55 | N/A |
| E2F8 | 4.55 | N/A |
| VDR | 4.55 | 0.45 |
| EPRS1 | 4.55 | -1.97 |
| RDH10 | 4.55 | N/A |
| TBX5 | 4.50 | 0.10 |
| SNCA | 4.49 | 2.29 |
| ZBTB17 | 4.40 | N/A |
| KLF6 | 4.38 | -0.49 |
| PRKG1 | 4.37 | 0.24 |
| Ige | 4.36 | -3.21 |
| PDGFB | 4.34 | -1.14 |
| Raf | 4.34 | -0.99 |
| STEAP3 | 4.33 | 3.00 |
| CCND1 | 4.30 | -2.72 |
| RFX2 | 4.28 | N/A |
| Tcf7 | 4.20 | 1.23 |
| MEF2C | 4.17 | 0.80 |
| TGFBR2 | 4.17 | -0.15 |
| TP63 | 4.15 | 0.65 |
| HRAS | 4.13 | 1.88 |
| BHLHE40 | 4.12 | 0.66 |
| YAP1 | 4.12 | -1.42 |
| MAPK1 | 4.10 | -2.35 |
| CASR | 4.08 | -0.56 |
| BMP2 | 4.08 | -2.20 |
| CTNNB1 | 4.06 | -2.08 |
| Tgf beta | 4.04 | -3.24 |
| YY1 | 4.00 | -0.92 |
| PSMB11 | 3.98 | 1.94 |
| HNF1B | 3.98 | 1.59 |
| DIO2 | 3.96 | -2.02 |
| EGR1 | 3.95 | -0.96 |
| miR-338-3p (miRNAs w/seed CCAGCAU) | 3.94 | 2.60 |
| E2F7 | 3.92 | N/A |
| MIR17HG | 3.87 | 2.53 |
| SLC16A2 | 3.87 | -0.54 |
| BMP10 | 3.85 | -0.30 |
| GSTO1 | 3.81 | -0.31 |
| Pkc(s) | 3.77 | -3.07 |
| STAT6 | 3.76 | -1.43 |
| LIPE | 3.75 | -0.22 |
| PITX2 | 3.75 | 0.69 |
| STAT5B | 3.75 | 0.34 |
| RETNLB | 3.74 | -3.15 |
| GNA15 | 3.74 | N/A |
| AHR | 3.69 | -0.67 |
| NFKB2 | 3.69 | 0.49 |
| PRKDC | 3.68 | 0.51 |
| FOXA2 | 3.67 | -0.40 |
| ERBB3 | 3.67 | 0.59 |
| LEF1 | 3.64 | 0.35 |
| Histone h4 | 3.63 | N/A |
| ATN1 | 3.61 | N/A |
| ERG | 3.60 | -2.63 |
| ADAMTS18 | 3.59 | -2.45 |
| ATOH7 | 3.56 | N/A |
| PCA3 | 3.56 | N/A |
| SUCNR1 | 3.55 | -2.24 |
| RFX1 | 3.55 | N/A |
| TCF3 | 3.53 | -0.08 |
| TGFB2 | 3.53 | -2.19 |
| IL6 | 3.52 | -1.89 |
| HSF1 | 3.52 | -2.09 |
| EGF | 3.51 | -1.65 |
| CDKN1A | 3.51 | 2.04 |
| FOS | 3.50 | -1.32 |
| Eldr | 3.48 | -1.41 |
| NKX3-1 | 3.48 | N/A |
| BMP6 | 3.46 | 0.44 |
| MYOC | 3.45 | N/A |
| CHUK | 3.44 | -1.27 |
| PCLAF | 3.43 | -1.43 |
| miR-17-5p (and other miRNAs w/seed AAAGUGC) | 3.42 | 2.10 |
| GDF11 | 3.42 | N/A |
| PI3K (complex) | 3.41 | -0.96 |
| PRKAA1 | 3.40 | 0.99 |
| ID3 | 3.36 | 1.55 |
| HIF1A | 3.35 | -2.58 |
| ITGA11 | 3.34 | -2.20 |
| Mek | 3.33 | -1.66 |
| THRB | 3.33 | -1.53 |
| let-7 | 3.31 | 2.58 |
| HDAC2 | 3.29 | 0.43 |
| ADAM19 | 3.27 | N/A |
| FGF12 | 3.27 | N/A |
| Snhg20 | 3.27 | N/A |
| ZNF395 | 3.27 | N/A |
| BRCA1 | 3.27 | 0.09 |
| CDH2 | 3.25 | -1.49 |
| ZNF217 | 3.25 | 2.45 |
| FGF2 | 3.24 | -0.71 |
| Notch | 3.19 | -2.27 |
| TCF7L1 | 3.18 | -0.79 |
| HNF1A | 3.18 | -2.82 |
| SRF | 3.18 | -2.62 |
| KLF4 | 3.18 | -1.82 |
| HMGA1 | 3.17 | -1.98 |
| AIF1 | 3.17 | -1.97 |
| KLF2 | 3.15 | 1.11 |
| miR-450a-5p (and other miRNAs w/seed UUUGCGA) | 3.14 | 2.45 |
| EHF | 3.14 | -0.75 |
| PPARG | 3.13 | -0.41 |
| miR-34a-5p (and other miRNAs w/seed GGCAGUG) | 3.13 | 2.54 |
| IL10RA | 3.13 | -2.27 |
| ERK | 3.13 | -1.51 |
| PTEN | 3.12 | -1.33 |
| IL17A | 3.11 | -0.60 |
| N6AMT1 | 3.11 | -3.05 |
| PKD1 | 3.10 | 1.50 |
| IL33 | 3.10 | -2.19 |
| CEBPB | 3.08 | -3.29 |
| MRTFA | 3.08 | -2.44 |
| ARID1A | 3.07 | -0.97 |
| THRA | 3.05 | 0.62 |
| EZH2 | 3.04 | -0.20 |
| PDGFRB | 3.04 | -1.15 |
| PDCD10 | 3.04 | 1.97 |
| IFNGR | 3.04 | N/A |
| ZNF521 | 3.04 | N/A |
| GDF2 | 3.04 | -3.16 |
| Rbp | 3.03 | N/A |
| AP1M1 | 3.03 | N/A |
| CDC23 | 3.03 | N/A |
| Rbx1 | 3.03 | N/A |
| MECP2 | 3.01 | -0.15 |
| SMAD7 | 3.00 | 1.11 |
| FSH | 3.00 | 0.42 |
| SOX2 | 2.99 | -0.44 |
| BRD4 | 2.99 | -3.68 |
| CNTF | 2.97 | 0.37 |
| NFKBIA | 2.96 | -2.14 |
| ADCYAP1 | 2.93 | -0.47 |
| BMP4 | 2.93 | -2.97 |
| NREP | 2.93 | -1.15 |
| ZFP91 | 2.93 | 2.00 |
| NFAT5 | 2.92 | -0.27 |
| CSF2 | 2.91 | -2.05 |
| HGF | 2.91 | -0.80 |
| AR | 2.90 | -2.21 |
| Immunoglobulin | 2.90 | 1.51 |
| C10orf99 | 2.89 | -0.82 |
| CREM | 2.87 | -0.73 |
| MAPK14 | 2.87 | -1.63 |
| IKBKG | 2.87 | -1.42 |
| SATB2 | 2.86 | 1.71 |
| PLA2G5 | 2.85 | N/A |
| EGR4 | 2.85 | N/A |
| OSM | 2.84 | -0.96 |
| miR-335-3p (miRNAs w/seed UUUUCAU) | 2.84 | 2.24 |
| HEY2 | 2.84 | 0.78 |
| NOTCH1 | 2.84 | -2.11 |
| DNMT3A | 2.83 | -0.99 |
| mir-8 | 2.83 | 1.79 |
| MAPK3 | 2.81 | -1.37 |
| INHA | 2.81 | 0.90 |
| NPM1 | 2.80 | -2.31 |
| miR-30c-5p (and other miRNAs w/seed GUAAACA) | 2.80 | 2.60 |
| S100A8 | 2.80 | 1.07 |
| GDNF | 2.80 | -1.07 |
| LIF | 2.79 | -0.89 |
| KDM3A | 2.78 | -0.20 |
| DSCAML1 | 2.76 | -3.00 |
| ERBB4 | 2.76 | 1.48 |
| IL6R | 2.76 | -0.90 |
| Brd4 | 2.75 | -2.54 |
| TAL1 | 2.75 | -1.00 |
| IL22 | 2.74 | -0.66 |
| VCAN | 2.73 | -0.83 |
| JAG1 | 2.72 | -1.52 |
| VGLL3 | 2.72 | -2.00 |
| MIRLET7 | 2.72 | 2.00 |
| miR-143-3p (and other miRNAs w/seed GAGAUGA) | 2.72 | 1.93 |
| BDNF | 2.72 | -1.40 |
| VHL | 2.71 | -0.80 |
| ROCK1 | 2.70 | -2.24 |
| INHBB | 2.70 | -1.23 |
| TP73 | 2.69 | -1.29 |
| LDB1 | 2.68 | -1.65 |
| SCN1B | 2.68 | N/A |
| MKX | 2.68 | N/A |
| EIF3E | 2.67 | 0.93 |
| SCD | 2.67 | 1.52 |
| TBX2 | 2.65 | -0.71 |
| DUSP10 | 2.63 | -1.15 |
| NUMB | 2.63 | -1.07 |
| PBRM1 | 2.63 | N/A |
| GATA6 | 2.63 | -1.58 |
| PI3K (family) | 2.60 | 0.65 |
| KLF11 | 2.60 | -0.84 |
| S100A9 | 2.59 | 1.39 |
| EGR2 | 2.59 | -3.26 |
| ASCL1 | 2.59 | -2.62 |
| PTF1A | 2.59 | -1.13 |
| LPL | 2.59 | -1.48 |
| FKBP10 | 2.59 | 2.65 |
| DPP4 | 2.57 | -1.07 |
| RB1 | 2.57 | -1.45 |
| LPXN | 2.56 | N/A |
| SUCO | 2.56 | N/A |
| TSIX | 2.56 | N/A |
| GSTA4 | 2.56 | N/A |
| miR-222-5p (miRNAs w/seed UCAGUAG) | 2.56 | N/A |
| miR-615-3p (miRNAs w/seed CCGAGCC) | 2.56 | N/A |
| REG3G | 2.56 | N/A |
| HOXD3 | 2.54 | -1.96 |
| AEBP1 | 2.53 | N/A |
| Arntl-Clock | 2.53 | N/A |
| SP3 | 2.51 | 0.20 |
| CR1L | 2.51 | 2.24 |
| RNA polymerase II | 2.48 | N/A |
| mir-21 | 2.48 | 1.02 |
| ADRA1D | 2.48 | N/A |
| miR-204-5p (and other miRNAs w/seed UCCCUUU) | 2.47 | 1.13 |
| mir-29 | 2.47 | 1.21 |
| SRA1 | 2.46 | -1.97 |
| CHEK1 | 2.46 | -0.85 |
| DSP | 2.46 | 1.98 |
| LOC105372576 | 2.45 | -1.40 |
| Calcineurin A | 2.45 | -0.76 |
| HHEX | 2.45 | N/A |
| EGFR | 2.45 | -1.53 |
| E2F3 | 2.45 | -0.28 |
| miR-29b-3p (and other miRNAs w/seed AGCACCA) | 2.44 | 2.90 |
| GATA4 | 2.44 | -0.46 |
| PTK2 | 2.44 | -1.85 |
| NEUROG1 | 2.44 | -0.38 |
| KRAS | 2.42 | 2.22 |
| Cxcl3 | 2.41 | N/A |
| MGAT1 | 2.41 | N/A |
| ARID4A | 2.41 | N/A |
| TGFBR1 | 2.39 | -2.95 |
| GNA13 | 2.39 | N/A |
| Alpha catenin | 2.36 | 2.38 |
| HAND2 | 2.36 | 1.70 |
| NFYB | 2.36 | N/A |
| INSR | 2.35 | 0.13 |
| TIMP3 | 2.35 | -0.82 |
| RARA | 2.33 | -0.52 |
| WWTR1 | 2.31 | -1.05 |
| PRDM1 | 2.31 | -0.12 |
| HYAL1 | 2.30 | -0.82 |
| DGCR8 | 2.30 | -0.92 |
| NRG1 | 2.30 | -1.85 |
| MBNL1 | 2.29 | N/A |
| FANCC | 2.29 | N/A |
| ATOH1 | 2.29 | N/A |
| NKX2-1 | 2.29 | -0.34 |
| SOX4 | 2.28 | -3.21 |
| IL1A | 2.27 | 0.18 |
| ARSA | 2.27 | N/A |
| UBE2K | 2.27 | N/A |
| ARID5B | 2.27 | N/A |
| Bc1 | 2.27 | N/A |
| CHRM4 | 2.27 | N/A |
| FDX2 | 2.27 | N/A |
| FDXR | 2.27 | N/A |
| miR-516a-3p (and other miRNAs w/seed GCUUCCU) | 2.27 | N/A |
| NNMT | 2.27 | N/A |
| RAPSN | 2.27 | N/A |
| CKS2 | 2.27 | N/A |
| GCN1 | 2.27 | N/A |
| IBSP | 2.27 | N/A |
| VSNL1 | 2.27 | N/A |
| EP300 | 2.27 | -1.44 |
| Creb | 2.26 | -1.69 |
| CLIC4 | 2.26 | 1.96 |
| CKAP2L | 2.26 | -1.63 |
| NEUROG3 | 2.25 | -2.41 |
| FABP1 | 2.24 | 0.00 |
| miR-18a-5p (and other miRNAs w/seed AAGGUGC) | 2.24 | 1.13 |
| GLI1 | 2.24 | -1.10 |
| CTF1 | 2.24 | -0.97 |
| NEUROG2 | 2.24 | N/A |
| mir-802 | 2.22 | 2.11 |
| RHO | 2.22 | 0.82 |
| BAX | 2.22 | -0.13 |
| MAP3K1 | 2.22 | -1.23 |
| ROCK | 2.22 | 0.00 |
| NSUN6 | 2.19 | -1.34 |
| FAS | 2.19 | 1.80 |
| S1PR4 | 2.18 | -0.64 |
| DCN | 2.18 | 0.48 |
| mir-223 | 2.18 | -0.31 |
| TNFRSF11B | 2.18 | -1.00 |
| Nppb | 2.18 | 2.00 |
| SOX1 | 2.16 | 1.89 |
| TRAF3IP2 | 2.16 | -0.11 |
| SMAD3 | 2.15 | -2.61 |
| ITGAV | 2.15 | -1.80 |
| THBS1 | 2.14 | 1.00 |
| LCN2 | 2.14 | -0.07 |
| DKK1 | 2.13 | 1.07 |
| IRS1 | 2.12 | -1.97 |
| PTPN11 | 2.12 | 1.96 |
| IL31 | 2.12 | 0.75 |
| RNF20 | 2.12 | -0.15 |
| beta-galactosidase | 2.12 | N/A |
| SPOP | 2.12 | N/A |
| LMO2 | 2.10 | -1.21 |
| LEP | 2.10 | -1.52 |
| SPDEF | 2.09 | 1.89 |
| CXCL5 | 2.09 | N/A |
| LAMC1 | 2.09 | N/A |
| LMNA | 2.08 | N/A |
| ERK1/2 | 2.08 | -1.73 |
| HDAC1 | 2.07 | 2.58 |
| CCR2 | 2.07 | -1.33 |
| LGALS3 | 2.07 | -1.42 |
| Rxr | 2.07 | N/A |
| GRIN3A | 2.07 | 1.00 |
| FGFR2 | 2.06 | -1.19 |
| CTSS | 2.06 | N/A |
| HEY1 | 2.06 | N/A |
| MEIS2 | 2.06 | -1.11 |
| DSCAM | 2.06 | -2.12 |
| IHH | 2.06 | -1.35 |
| BRD8 | 2.06 | N/A |
| ADAMTS1 | 2.06 | N/A |
| CMA1 | 2.06 | N/A |
| COL11A1 | 2.06 | N/A |
| Collagen type V | 2.06 | N/A |
| ENTPD5 | 2.06 | N/A |
| Foxe3 | 2.06 | N/A |
| GLS | 2.06 | N/A |
| MIR143-145a | 2.06 | N/A |
| RFXANK | 2.06 | N/A |
| TMPRSS4 | 2.06 | N/A |
| TRHR | 2.06 | N/A |
| ARLNC1 | 2.06 | N/A |
| HOXD12 | 2.06 | N/A |
| SNHG3 | 2.06 | N/A |
| NKX2-5 | 2.05 | -1.62 |
| DLK1 | 2.05 | 1.09 |
| MSX2 | 2.05 | N/A |
| CIITA | 2.05 | 1.11 |
| SLC9A3R1 | 2.05 | 0.45 |
| Nr1h | 2.04 | 0.19 |
| GABPA | 2.04 | -0.76 |
| NOTCH3 | 2.03 | -1.19 |
| PIAS1 | 2.03 | -1.32 |
| ELK1 | 2.03 | 0.34 |
| TAF4 | 2.03 | 1.66 |
| NCOA3 | 2.03 | -1.01 |
| CDKN2A | 2.02 | 2.46 |
| KMT2D | 2.01 | -0.18 |
| BRAF | 2.01 | N/A |
| EDNRA | 2.00 | N/A |
| SMC3 | 2.00 | N/A |
| YWHAQ | 2.00 | N/A |
| PRKAR2B | 2.00 | -1.98 |
| NPR1 | 2.00 | 1.96 |
| mir-204 | 2.00 | -1.00 |
| BNIP3L | 2.00 | 1.63 |

Only significantly enriched upstream regulators are displayed (p ≤ 0.01 (-log(P-value) ≥ 2)). The Z-score indicates the predicted regulation of an upstream regulator: Z-score ≤ -2 indicates relevant downregulation of the upstream regulator; Z-score ≥ 2 indicates relevant upregulation of the upstream regulator.

**Supplementary table S14: Upstream regulator analysis based on hippocampal gene expression in HFD group (8 months old) vs Aging-Chow group (8 months old).**

| **8 month-old HFD vs 8 month-old Chow** | | |
| --- | --- | --- |
| Upstream regulator | -log(P) | Z-score |
| SPI1 | 14.07 | 3.54 |
| GRN | 12.26 | -2.00 |
| TCL1A | 10.88 | N/A |
| Immunoglobulin | 10.87 | -0.85 |
| MAPT | 10.69 | N/A |
| KDM1A | 10.36 | N/A |
| HNRNPU | 9.58 | -3.00 |
| USP22 | 9.24 | 0.95 |
| PNPT1 | 8.98 | -2.83 |
| IFNG | 8.89 | 4.11 |
| B4GALNT1 | 7.89 | -2.22 |
| ITPR2 | 7.59 | 2.81 |
| G protein alpha i | 7.54 | 2.83 |
| ST8SIA1 | 6.71 | -2.22 |
| IL10 | 6.36 | 2.01 |
| JAK1 | 6.30 | 0.85 |
| TNF | 6.21 | 3.27 |
| CSF1 | 6.21 | 2.03 |
| TRIM24 | 6.15 | -2.80 |
| TREX1 | 6.14 | -2.56 |
| STAT1 | 6.13 | 3.23 |
| RNASEH2B | 6.06 | -2.79 |
| Interferon alpha | 5.91 | 3.22 |
| CNTF | 5.84 | 2.33 |
| SLC15A4 | 5.56 | 2.80 |
| Ifnar | 5.44 | 2.58 |
| miR-219a-5p (and other miRNAs w/seed GAUUGUC) | 5.29 | N/A |
| STAG2 | 5.14 | -2.23 |
| CCL20 | 5.14 | 2.24 |
| PCGF6 | 5.13 | 0.12 |
| DUSP11 | 5.04 | -2.00 |
| BHLHE40 | 5.04 | 3.32 |
| CSF2 | 4.99 | 2.34 |
| CYP2E1 | 4.88 | 1.99 |
| KRAS | 4.76 | N/A |
| IRF7 | 4.74 | 2.79 |
| NR1H3 | 4.65 | N/A |
| DRD2 | 4.62 | 1.66 |
| LDLR | 4.53 | N/A |
| IFNA2 | 4.45 | 2.57 |
| SOCS1 | 4.40 | -2.43 |
| AGT | 4.39 | 3.76 |
| GSDMB | 4.39 | N/A |
| Irgm1 | 4.38 | -2.43 |
| DNASE2 | 4.34 | N/A |
| TYROBP | 4.30 | N/A |
| IL4 | 4.26 | -0.51 |
| Ttc39aos1 | 4.24 | -2.22 |
| PTGER4 | 4.14 | -1.96 |
| SPRY2 | 4.10 | -1.30 |
| APP | 4.09 | 1.78 |
| NCSTN | 4.08 | -2.00 |
| PTPN11 | 4.06 | -1.43 |
| PTPN6 | 4.05 | -0.28 |
| IL6 | 4.04 | 0.66 |
| RNASEL | 4.03 | N/A |
| ELOVL3 | 4.02 | 2.24 |
| STAT3 | 3.95 | 0.39 |
| KLK1 | 3.95 | N/A |
| ETV5 | 3.93 | -0.64 |
| TGM2 | 3.85 | 2.80 |
| IL21 | 3.81 | 1.61 |
| FZD9 | 3.78 | N/A |
| NKX2-1 | 3.67 | N/A |
| SENP3 | 3.66 | 2.00 |
| Hbb-b2 | 3.66 | 1.96 |
| PSEN1 | 3.57 | -2.19 |
| ADIPOQ | 3.57 | -1.34 |
| HRG | 3.56 | N/A |
| IRF8 | 3.56 | -0.07 |
| ACKR2 | 3.55 | -2.00 |
| RNASEH2A | 3.49 | N/A |
| Ige | 3.48 | 2.98 |
| Pka | 3.48 | 2.22 |
| Hbb-b1 | 3.47 | 2.20 |
| SNCA | 3.46 | 1.34 |
| MYD88 | 3.45 | 1.78 |
| Tcf7 | 3.40 | -1.49 |
| KLF6 | 3.38 | 2.16 |
| IRF3 | 3.38 | 2.39 |
| IL33 | 3.33 | 0.98 |
| CX3CL1 | 3.29 | 2.22 |
| EIF4E | 3.29 | 1.89 |
| GPR174 | 3.28 | -0.45 |
| TNFSF11 | 3.28 | 1.91 |
| NRAS | 3.28 | -1.98 |
| TLR7 | 3.27 | 2.43 |
| ZBTB10 | 3.23 | 2.45 |
| CLEC12A | 3.22 | N/A |
| CSF3 | 3.20 | 2.19 |
| STAT2 | 3.19 | N/A |
| SIRT1 | 3.17 | -3.15 |
| CITED2 | 3.17 | -2.62 |
| ETS1 | 3.16 | 2.20 |
| CEBPB | 3.16 | -0.98 |
| TBK1 | 3.12 | N/A |
| APOE | 3.11 | -2.77 |
| PPARD | 3.10 | 0.36 |
| ELF1 | 3.07 | N/A |
| TRIM14 | 3.07 | N/A |
| CCR2 | 3.06 | 2.19 |
| KIT | 3.05 | N/A |
| IRAK3 | 3.03 | N/A |
| DIO3 | 3.03 | -0.82 |
| IL13 | 3.00 | 0.08 |
| IL10RA | 3.00 | -0.71 |
| AIRE | 2.98 | N/A |
| TLR9 | 2.96 | 1.00 |
| ABCC1 | 2.95 | N/A |
| TGFBR1 | 2.95 | -2.24 |
| USP8 | 2.94 | -1.98 |
| JAK1/2 | 2.94 | 2.00 |
| PRL | 2.93 | 1.51 |
| C1QA | 2.91 | N/A |
| TMEM120A | 2.84 | N/A |
| LY86 | 2.84 | N/A |
| SMARCA5 | 2.82 | -2.24 |
| PLCG2 | 2.81 | -1.00 |
| AIM2 | 2.80 | N/A |
| Ap2 | 2.80 | N/A |
| NR5A2 | 2.78 | 0.79 |
| NONO | 2.77 | 2.22 |
| PRDM16 | 2.73 | N/A |
| Tnf (family) | 2.72 | -1.07 |
| MEF2A | 2.71 | 2.00 |
| IRF2 | 2.71 | -0.15 |
| RUNX1 | 2.68 | N/A |
| JUND | 2.67 | N/A |
| mir-21 | 2.66 | -0.71 |
| EEF1A2 | 2.66 | N/A |
| C3 | 2.65 | 0.75 |
| STING1 | 2.64 | 2.17 |
| mir-155 | 2.63 | -2.19 |
| IRF1 | 2.62 | 1.39 |
| IFNL1 | 2.62 | 1.97 |
| CGAS | 2.60 | N/A |
| RARA | 2.58 | -1.00 |
| CASP4 | 2.57 | N/A |
| TP53 | 2.57 | 0.30 |
| LMO2 | 2.57 | -1.89 |
| MIF | 2.54 | -2.20 |
| ILF3 | 2.53 | 2.00 |
| FLT3 | 2.52 | N/A |
| DUSP1 | 2.51 | -2.22 |
| LDB1 | 2.50 | -1.89 |
| PRDM1 | 2.50 | -1.13 |
| IFNAR2 | 2.49 | N/A |
| JAK2 | 2.47 | N/A |
| IFNB1 | 2.46 | 2.16 |
| EHF | 2.46 | -0.25 |
| DYSF | 2.46 | N/A |
| ADAR | 2.45 | N/A |
| Hsp27 | 2.44 | N/A |
| OLR1 | 2.44 | N/A |
| STAT6 | 2.42 | -0.12 |
| PPARA | 2.42 | -0.38 |
| HOXA3 | 2.41 | N/A |
| IFNAR1 | 2.41 | N/A |
| NCOR1 | 2.40 | N/A |
| UCP1 | 2.37 | -2.19 |
| RELA | 2.37 | 2.16 |
| PSEN2 | 2.35 | -1.98 |
| IFIH1 | 2.34 | N/A |
| CSK | 2.33 | N/A |
| FCGR1A | 2.33 | N/A |
| miR-125b-5p (and other miRNAs w/seed CCCUGAG) | 2.29 | -1.97 |
| CYP19A1 | 2.29 | N/A |
| BAX | 2.29 | N/A |
| ANXA1 | 2.29 | N/A |
| TNK1 | 2.28 | N/A |
| FGF10 | 2.27 | N/A |
| CEBPE | 2.27 | N/A |
| FCGR2A | 2.27 | N/A |
| HIVEP1 | 2.27 | N/A |
| IFN Beta | 2.25 | 2.42 |
| TP73 | 2.25 | -0.33 |
| IRF9 | 2.25 | N/A |
| INSIG1 | 2.24 | N/A |
| RARB | 2.23 | -0.65 |
| SOD3 | 2.23 | N/A |
| HRAS | 2.22 | 0.71 |
| CLEC11A | 2.21 | N/A |
| RGS10 | 2.21 | N/A |
| TGFB1 | 2.20 | 2.72 |
| PTPRM | 2.19 | N/A |
| 4930481B07Rik | 2.19 | N/A |
| ARR3 | 2.19 | N/A |
| Ctla2a/Ctla2b | 2.19 | N/A |
| FBXO46 | 2.19 | N/A |
| GPLD1 | 2.19 | N/A |
| RP11_750H95 | 2.19 | N/A |
| TSKU | 2.19 | N/A |
| ZNF433 | 2.19 | N/A |
| CLEC4A | 2.19 | N/A |
| CSF3R | 2.18 | N/A |
| FOXO4 | 2.17 | -2.00 |
| GIP | 2.17 | N/A |
| UBD | 2.14 | N/A |
| BID | 2.14 | N/A |
| MAFB | 2.12 | 1.00 |
| OSMR | 2.11 | N/A |
| MVP | 2.10 | N/A |
| MSC | 2.09 | N/A |
| PSMB11 | 2.08 | -2.00 |
| IL1B | 2.07 | 1.98 |
| GSTO1 | 2.07 | N/A |
| SFTPD | 2.06 | N/A |
| Pde4 | 2.02 | N/A |
| Cyp2c70 | 2.02 | N/A |
| FLI1 | 2.02 | N/A |

Only significantly enriched upstream regulators are displayed (p ≤ 0.01 (-log(P-value) ≥ 2)). The Z-score indicates the predicted regulation of an upstream regulator: Z-score ≤ -2 indicates relevant downregulation of the upstream regulator; Z-score ≥ 2 indicates relevant upregulation of the upstream regulator.

**Supplementary table S15: Correlations between cognition and parameters related to brain structure and function**

**
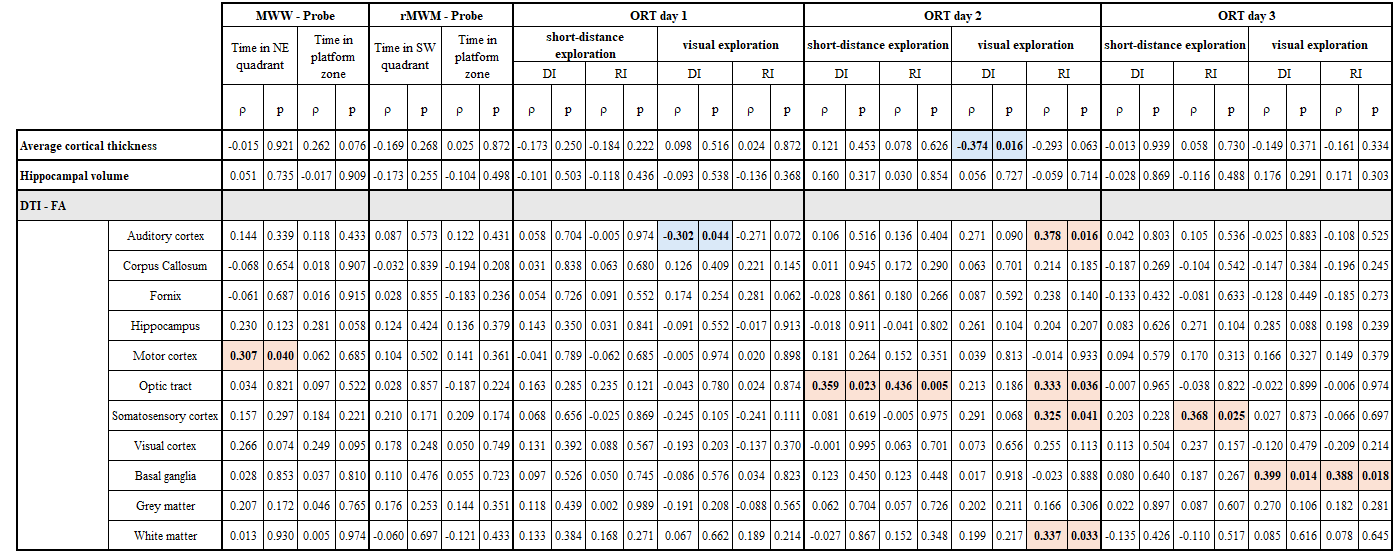
**


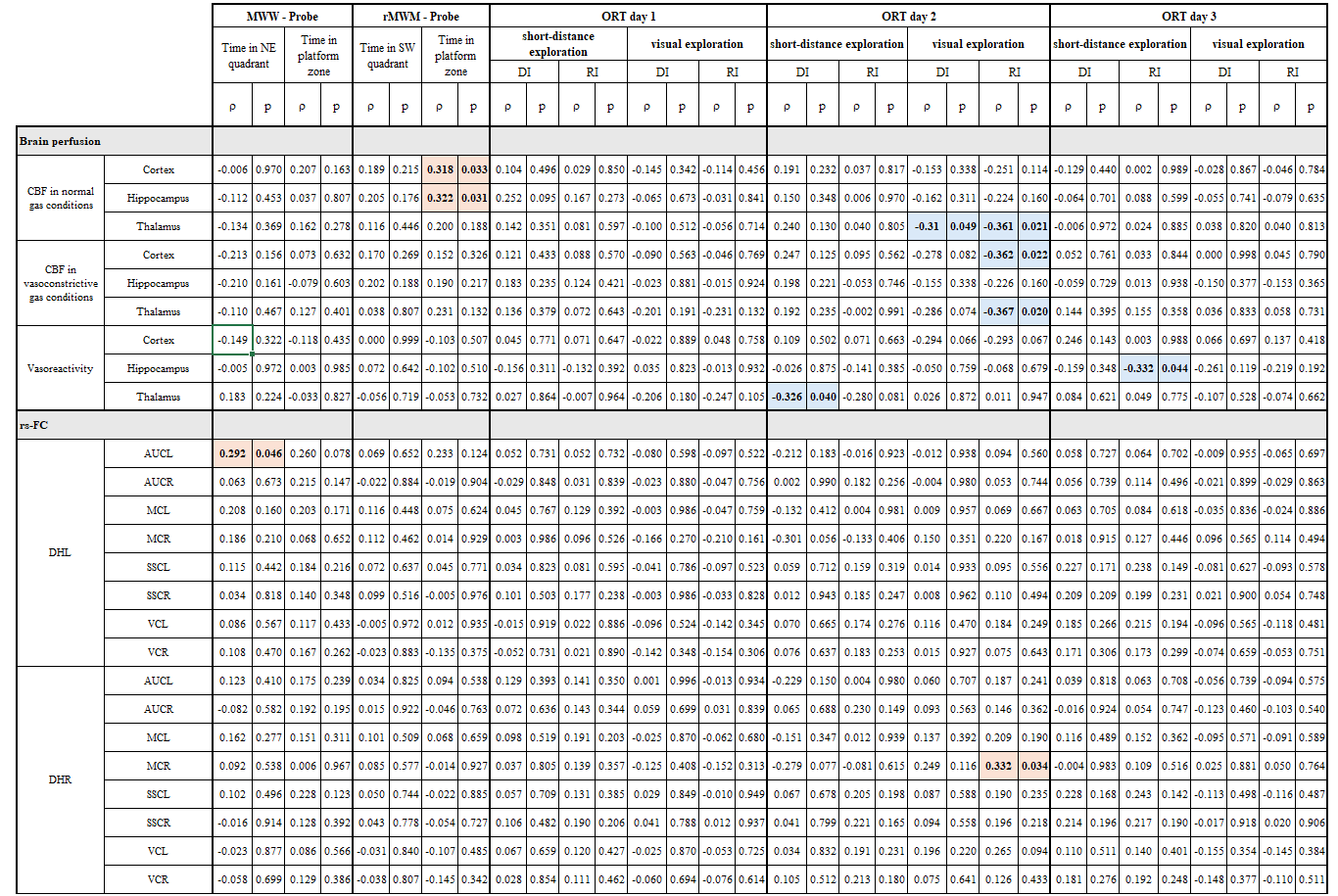


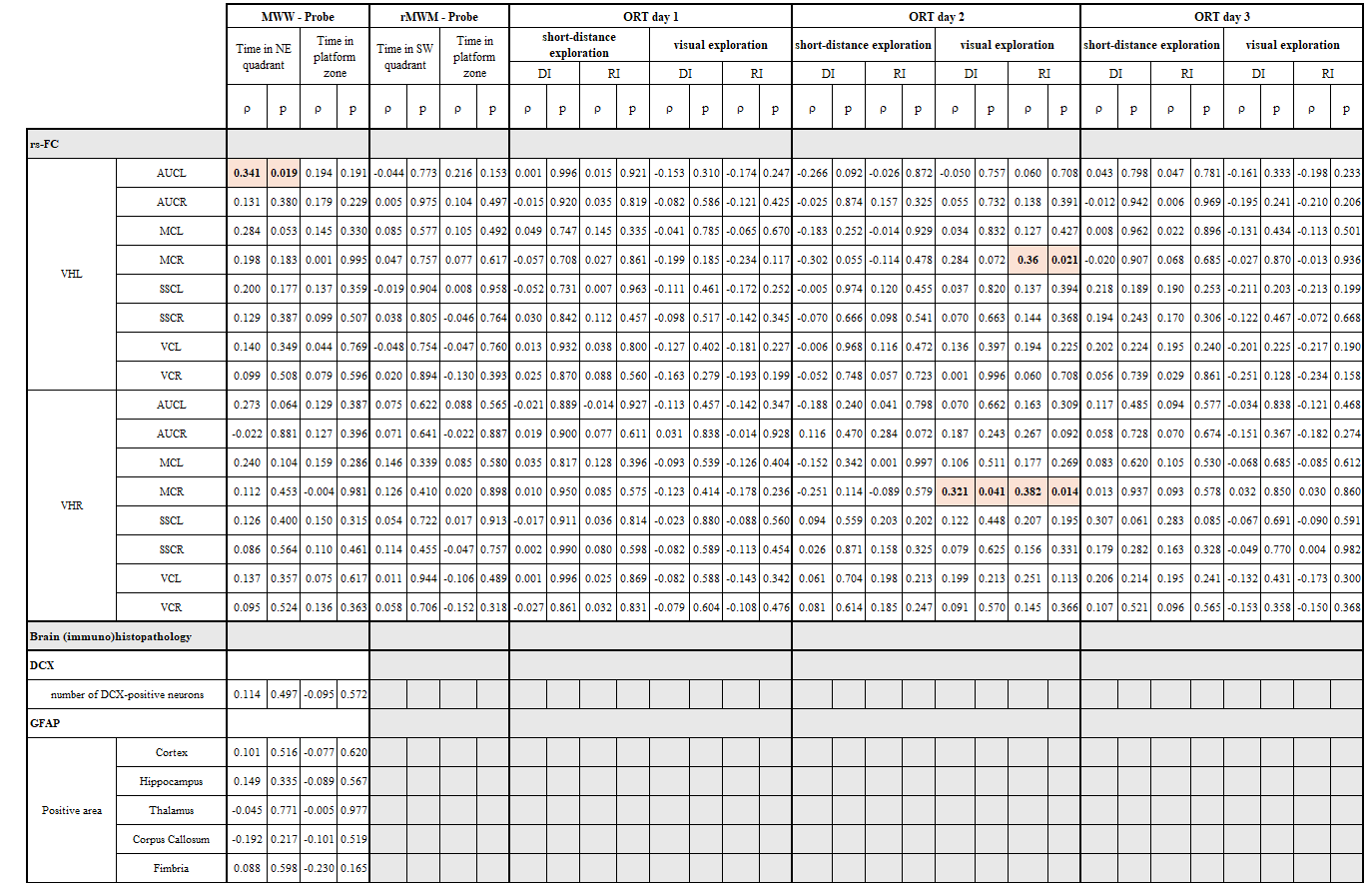


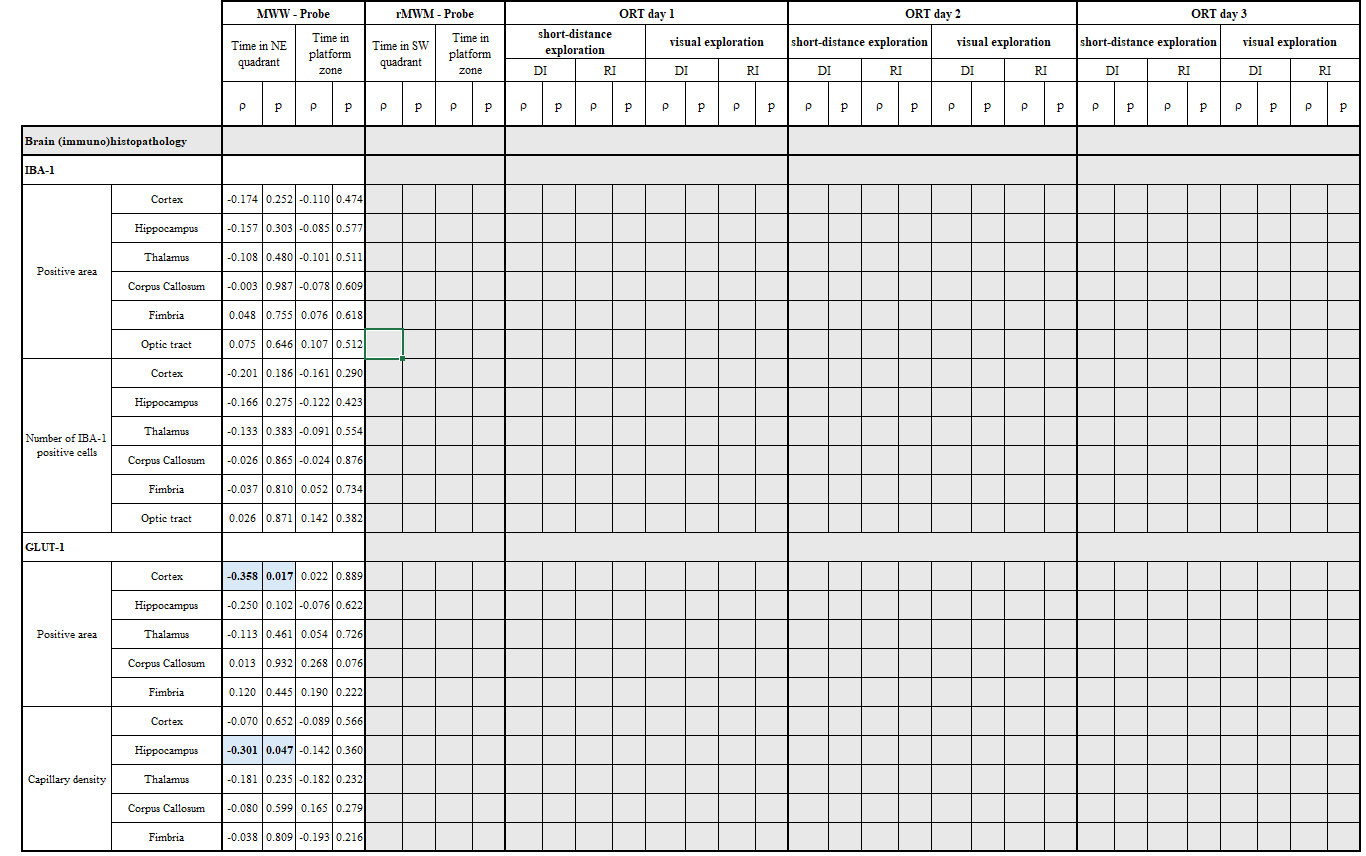


Significant associations (p ≤ 0.05) are indicated in bold. Correlation coefficients ρ < 0 indicate negative associations (shown in blue) while ρ > 0 indicate positive associations (shown in orange). Abbreviations for resting-state functional connectivity (rs-FC): (DH) dorsal hippocampus; (VH) ventral hippocampus; (AUC) auditory cortex; (MC) motor cortex; (SSC) somatosensory cortex; (VC) visual cortex in left (L) and right (R) hemispheres. Other abbreviations: (DCX) doublecortin; (DI) discrimination index; (GFAP) glial fibrillary acidic protein; (GLUT-1) glucose transporter 1; (IBA-1) ionized calcium-binding adapter molecule 1; (NE) North-East; ((r)MWM) (reverse) Morris Water Maze; (ORT) Object Recognition Test; (RI) recognition index; (SW) South-West.

## References

[1] K.J. Lohkamp, A.M. van den Hoek, G. Solé-Guardia, M. Lisovets, T. Alves Hoffmann, K. Velanaki, B. Geenen, V. Verweij, M.C. Morrison, R. Kleemann, M. Wiesmann, A.J. Kiliaan, The Preventive Effect of Exercise and Oral Branched-Chain Amino Acid Supplementation on Obesity-Induced Brain Changes in Ldlr−/−.Leiden Mice, 2023. https://doi.org/10.3390/nu15071716.

[2] A.C. Tengeler, E. Gart, M. Wiesmann, I.A.C. Arnoldussen, W. van Duyvenvoorde, M. Hoogstad, P.J. Dederen, V. Verweij, B. Geenen, T. Kozicz, R. Kleemann, M.C. Morrison, A.J. Kiliaan, Propionic acid and not caproic acid, attenuates nonalcoholic steatohepatitis and improves (cerebro) vascular functions in obese Ldlr−/−.Leiden mice, FASEB J. 34 (2020) 9575–9593. https://doi.org/10.1096/fj.202000455R.

[3] I.A.C. Arnoldussen, M.C. Morrison, M. Wiesmann, J.A. van Diepen, N. Worms, M. Voskuilen, V. Verweij, B. Geenen, N.P. Gualdo, L. van der Logt, G. Gross, R. Kleemann, A.J. Kiliaan, Milk fat globule membrane attenuates high fat diet-induced neuropathological changes in obese Ldlr−/−.Leiden mice, Int. J. Obes. 46 (2022) 342–349. https://doi.org/10.1038/s41366-021-00998-w.

[4] G. Paxinos, K.B.J. Franklin, The Mouse Brain in Stereotaxic Coordinates, 3rd ed., Academic Press: Cambridge, n.d.

[5] A.L. Alexander, J.E. Lee, M. Lazar, A.S. Field, Diffusion Tensor Imaging of the Brain, Neurotherapeutics 4 (2007) 316–329. https://doi.org/10.1016/j.nurt.2007.05.011.

[6] M. Wiesmann, V. Zerbi, D. Jansen, D. Lütjohann, A. Veltien, A. Heerschap, A.J. Kiliaan, Hypertension, cerebrovascular impairment, and cognitive decline in aged AβPP/PS1 mice, Theranostics 7 (2017) 1277–1289. https://doi.org/10.7150/thno.18509.

[7] V. Zerbi, M. Wiesmann, T.L. Emmerzaal, D. Jansen, M. Van Beek, M.P.C. Mutsaers, C.F. Beckmann, A. Heerschap, A.J. Kiliaan, Resting-state functional connectivity changes in aging apoe4 and apoe-ko mice, J. Neurosci. 34 (2014) 13963–13975. https://doi.org/10.1523/JNEUROSCI.0684-14.2014.
